# Adaptive plasticity of natural interleukin-35-induced regulatory T cells (Tr35) that are required for T-cell immune regulation

**DOI:** 10.7150/thno.90608

**Published:** 2024-05-05

**Authors:** Xundong Wei, Jianhua Zhang, Jian Cui, Wei Xu, Gang Zhao, Chang Guo, Wei Yuan, Xuyu Zhou, Jie Ma

**Affiliations:** 1 Center of Biotherapy, Beijing Hospital, National Center of Gerontology; Institute of Geriatric Medicine, Chinese Academy of Medical Sciences, Beijing 100730, P.R. China.; 2 CAS Key Laboratory of Pathogenic Microbiology and Immunology, Institute of Microbiology, Chinese Academy of Sciences (CAS), Beijing 100101, P.R. China.; 3 Ruikang Hospital Affiliated to Guangxi University of Chinese Medicine, Guangxi Nanning, P.R. China.; 4 Department of General Surgery, Beijing Hospital, National Center of Gerontology; Institute of Geriatric Medicine, Chinese Academy of Medical Sciences, Beijing, 100730, P.R. China.; 5 National Cancer Center/National Clinical Research Center for Cancer/Cancer Hospital, Chinese Academy of Medical Sciences and Peking Union Medical College, Beijing, 100021, P.R. China.; 6 Savaid Medical School, University of Chinese Academy of Sciences, Beijing 101408, P.R. China.

**Keywords:** induced Treg, interleukin-35, Tr35 trajectory, transgenic reporter mice

## Abstract

**Background:** IL-35 potently inhibits immune responses both *in vivo* and *in vitro*. However, the specific characteristics of IL-35-producing cells, including their developmental origin, cellular phenotype, and function, are unknown.

**Methods:** By using a novel IL-35 reporter mouse (Ebi3-Dre-Thy1.1) and double transgenic fate-mapping reporter mice (35EbiT-Rosa26-rox-tdTomato reporter mice or Foxp3 fate-mapping system), we tracked and analyzed the differentiation and developmental trajectories of Tr35 cells *in vivo*. And then we investigated the therapeutic effects of OVA-specific Tr35 cells in an OVA-induced allergic airway disease model.

**Results:** We identified a subset of cells, denoted Tr35 cells, that secrete IL-35 but do not express Foxp3. These cells have high expression of molecules associated with T-cell activation and can inhibit T-cell proliferation *in vitro*. Our analyses showed that Tr35 cells are a distinct subpopulation of cells that are independent of Tr1 cells. Tr35 cells exhibit a unique gene expression profile and tissue distribution. The presence of Thy1.1 (Ebi3) expression in Tr35 cells indicates their active secretion of IL-35. However, the proportion of ex-Tr35 cells (Thy1.1^-^) is significantly higher compared to Tr35 cells (Thy1.1^+^). This suggests that Tr35 cells possess the ability to regulate IL-35 expression rapidly *in vivo*. Tr35 cells downregulated the expression of the inflammatory cytokines IL-4, IFN-γ and IL-17A. However, once Tr35 cells lost IL-35 expression and became exTr35 cells, the expression of inflammatory cytokines was upregulated. Importantly, our findings indicate that Tr35 cells have therapeutic potential. In an OVA-induced allergic airway disease mouse model, Tr35 cell reinfusion significantly reduced airway hyperresponsiveness and histopathological airway and lung inflammation.

**Conclusions:** We have identified a subset of Tregs, Tr35 cells, that are distinct from Tr1 cells. Tr35 cells can dynamically regulate the secretion of inflammatory cytokines by controlling IL-35 expression to regulate inflammatory immune responses.

## Introduction

Regional immune tolerance depends on the release of soluble inhibitory cytokines such as interleukin (IL)-10, IL-35 and TGF-β 1. IL-35 is the most recently discovered member of the IL-12 cytokine family 2, which includes IL-12, IL-23, IL-27, and IL-35. The members of the IL-12 family are heterodimeric proteins composed of an α chain (p19, p28, or p35) and a β chain [p40 or Epstein-Barr virus-induced gene 3 (Ebi3)]. IL-35 is composed of p35 (IL-12a) and Ebi3 3. Devergne *et al*. were the first to report that p35 interacts with Ebi3 to form a heterodimer and speculated that this heterodimer might have an immunomodulatory function based on the expression of Ebi3 in immune cell-containing tissues 4. Unlike other members of the IL-12 family, IL-35 is mainly produced by regulatory T cells (Tregs) and rarely secreted by antigen-presenting cells (APCs) under immune equilibrium.

Specific knockdown of Ebi3 or p35 in Tregs significantly attenuates the inhibitory activity of these cells *in vitro* and *in vivo* and eliminates their ability to effectively control the occurrence of colitis in a model of enteritis 5. A study using Ebi3^-/-^ IL-12p35^-/-^ knockout mice demonstrated that IL-35 reduces inflammation in models of autoimmune diseases and chronic inflammation by inhibiting effector T cells 6. IL-35 has been shown to play immunosuppressive roles in inflammatory enteritis (inflammatory bowel disease, IBD), experimental autoimmune encephalomyelitis (EAE), collagen-induced arthritis (CIA), and myocarditis 7-9. IL-35 alleviates the symptoms of enteritis by downregulating the number of Th1 and Th17 cells in the intestinal mucosal immune microenvironment 10.

High expression of IL-35 in mouse pancreatic β-cells inhibits the infiltration of CD4^+^ and CD8^+^ T cells in pancreatic islets, inhibits T-cell proliferation, and provides long-term protection against autoimmune diabetes 11. IL-35 overexpression also has protective effects on graft versus host disease (GvHD), viral myocarditis, and atherosclerosis 12,13. In a mouse model of allergic airway disease, overexpression of IL-35 or IL-35 protein factors significantly decreases the levels of macrophages, eosinophils, allergen-specific lymphocytes, neutrophils, and cytokines (IL-4, IL-5, IL-13, IL-17, etc.) in alveolar lavage fluid, airway hyperresponsiveness and lung inflammation 14,15. Whitehead *et al*. reported that mice with defects in the genes for the subunits of IL-35 (i.e., *Ebi3* and *p35*) exhibit obvious airway inflammation and airway hyperresponsiveness after a single ovalbumin (OVA) challenge, while mice without gene defects have low airway responsiveness 16. Recent studies have also implicated IL-35 in the immune pathogenesis of asthma. Serum levels of IL-35 are significantly lower in children and adults with asthma than in healthy controls and decrease as the severity of asthma increases 17-20. These data present a proof of principle that IL-35 could be used as a potent regulator of inflammatory diseases, and further research is needed to establish its efficacy.

In addition to directly inhibiting the effector T-cell response, IL-35 induces immune tolerance to infectious diseases and tumors, and IL-35-producing Tregs induced by IL-35 have become a new hotspot in immune research 21-23. Collison *et al*. first discovered that treating conventional T cells (Tconvs, CD4^+^Foxp3^-^) with IL-35 during *in vitro* culture induces the differentiation of a new class of inhibitory T cells that they denoted "iTr35 cells" 22. Although iTr35 cells do not express the Treg-specific transcription factor Foxp3, they have a strong immunosuppressive function 24. Antibody blocking assays have shown that iTr35 cells exert their immunosuppressive effects through IL-35 rather than IL-10 or TGF-β *in vitro*. Collison *et al*. also confirmed that iTr35 cells effectively inhibit the pathogenesis of experimental spontaneous encephalomyelitis (EAE) in a mouse model of EAE 22. However, the current understanding of iTr35 cells is limited to those induced in culture *in vitro*. Unlike IL-10-producing Tr1 (CD4^+^Foxp3^-^IL-10^+^) cells, the characteristics of iTr35 cells, including their developmental origin, cellular phenotype, and specific function, are unknown. This is largely due to the rapid secretion of IL-35 and the lack of tools for isolating pure populations of iTr35 cells generated *in vivo*. More research may be needed to fully understand these cells.

In our previous work, we utilized a novel IL-35 reporter mouse (Ebi3-Dre-Thy1.1) to identify Ebi3-expressing cells based on the surface expression of Thy1.1. We observed an effector IL-35-Treg subset, which predominantly produce IL-35 and express intermediate levels of ICOS and CCR7, preferentially localize to the T cells zone of secondary lymphoid organs, play a role in damping the anti-tumor response 25. We also observed a subset of cells *in vivo* that do not express Foxp3 but secrete IL-35. These cells have high expression of molecules related to T-cell activation, such as CD44, ICOS, CD69, and PD1, and inhibit T-cell proliferation *in vitro*. To confirm our findings, we utilized the Foxp3 fate-mapping system 26 to examine Thy1.1 expression in various Treg populations with different Foxp3 expression levels. Our results showed that iTr35 cells generated *in vivo* (referred to hereafter as Tr35 cells to distinguish them from cells generated *in vitro*) are distinct from exTregs (CD4^+^CD25^low^Foxp3^low^ T cells) and are formed by the antigen stimulation of Tconvs in the absence of Tregs. We also determined that Tr35 cells are distinct from Tr1 cells and have a unique profile of surface molecule, chemokine receptor, and gene expression and tissue distribution.

In this study, using double transgenic fate-mapping reporter mice (35EbiT-Rosa26-rox-tdTomato reporter mice), we tracked and analyzed the differentiation and developmental trajectories of Tr35 cells *in vivo*. We found that the proportion of cells expressing IL-35 (Ebi3) was lower in Tr35 cells than in IL-35-Treg cells, indicating that Tr35 cells regulate the expression of IL-35 more rapidly *in vivo*. In addition, the expression of the inflammatory cytokines IFN-γ (interferon-gamma) and IL-17A was downregulated in Tr35 cells. However, once Tr35 cells lost IL-35 expression and became exTr35 cells, the expression of inflammatory cytokines was upregulated. Moreover, exTr35 cells partially differentiated into IL-10-expressing Tr1 cells, indicating that a subset of the cells can undergo a switch toward the secretion of suppressive cytokines. Furthermore, the results of IL-35 fate-mapping studies suggested that Tr35 cells may be transiently associated with the activation of Th cells (Th1, Th2, Th17) rather than a dedicated Tr35 lineage. In an OVA-induced allergic airway disease mouse model, we found that reinfusion of Tr35 cells significantly reduced airway hyperresponsiveness and histopathological airway and lung inflammation. In summary, we have identified a subset of Tregs, Tr35 cells, that are distinct from Tr1 cells. Tr35 cells can dynamically regulate the secretion of inflammatory cytokines by controlling IL-35 expression to regulate inflammatory immune responses.

## Results

### Phenotypic analysis of Tr35 cell subsets

**Figure 1:**
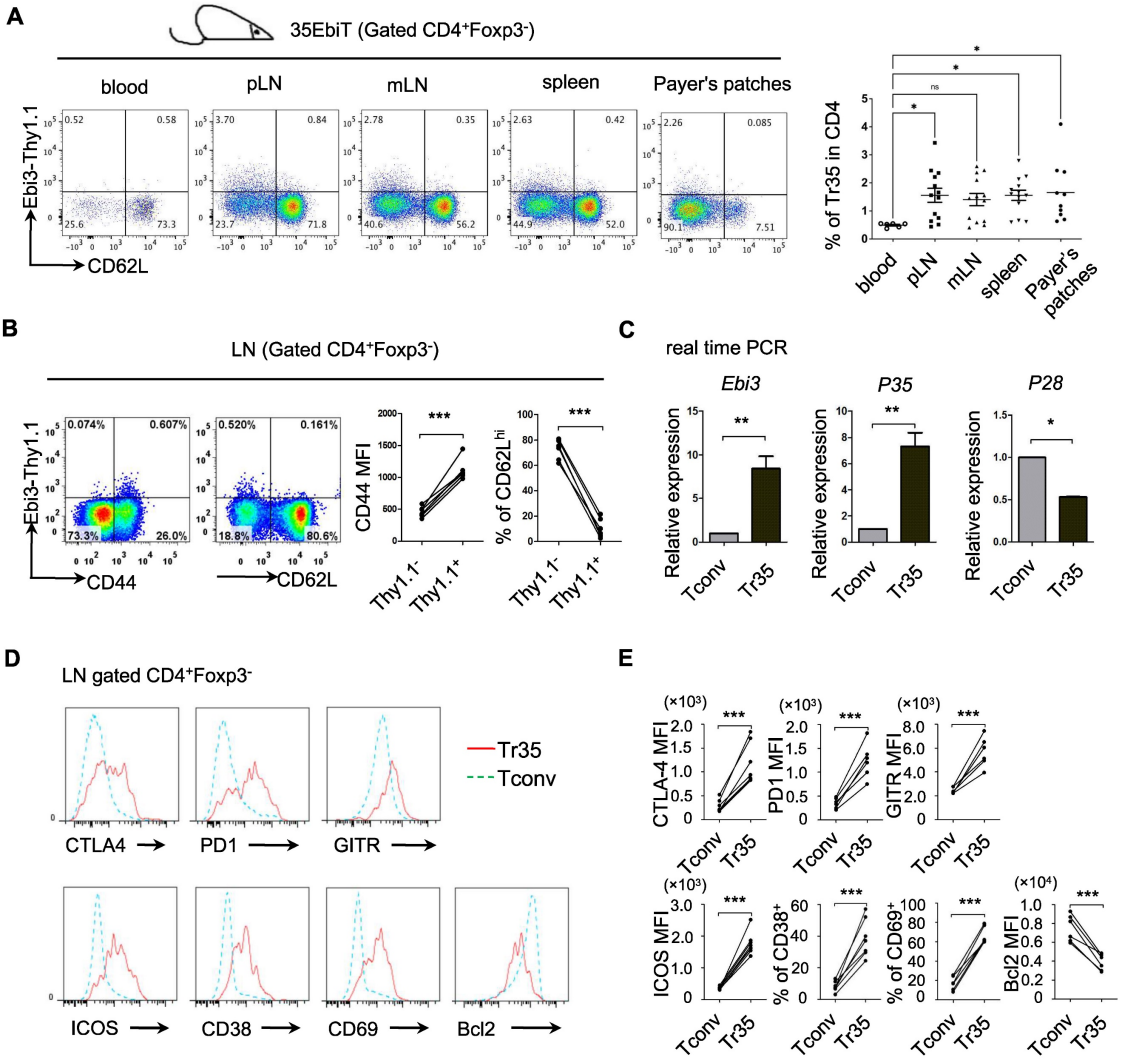
** Phenotypic analysis of Tr35 cell subsets. (A)** Flow cytometric analysis of the proportion of Tr35 cells in various tissues and blood of 35EbiT transgenic mice. **(B)** Flow cytometry analysis of CD4^+^ T cells isolated from the lymph nodes of 35EbiT transgenic mice. The numbers in the plots indicate the percentage of cells in each quadrant (left). The expression levels of CD44 and CD62L on Tr35 cells (gated on CD4^+^Foxp3^-^Thy1.1^+^ T cells) and Tconvs (gated on CD4^+^Foxp3^-^Thy1.1^-^ T cells) from the lymph nodes of 35EbiT transgenic mice were analyzed (right). **(C)** Quantitative RT-PCR analysis of *Ebi3, P35,* and *P28* mRNA expression in sorted Thy1.1^+^ (CD4^+^GFP^-^CD62L^low^Thy1.1^+^) and Thy1.1^-^ (CD4^+^GFP^-^CD62L^hi^Thy1.1^-^) cells from the lymph nodes of 35EbiT × Foxp3-GFP KI dual reporter mice. The results are presented relative to the expression of GAPDH. **(D)** Flow cytometry analysis of CD4^+^ T cells isolated from the lymph nodes of 35EbiT transgenic mice in the steady state. The cells were stained with antibodies against CD4, Thy1.1, Foxp3, and other cell surface markers. The histogram plots were gated on Foxp3^-^Thy1.1^-^ (blue line) and Foxp3^-^Thy1.1^+^ (red line) cells. **(E)** Mean fluorescence intensity (MFI) and frequencies of the indicated markers; cumulative results from three independent experiments, respectively, are shown. The data shown are typical results from three similar experiments, n=5. The lines connecting the points represent statistical analyses comparing different cell populations within the same mouse. The error bars represent the mean ± SEM. *p < 0.05, **p < 0.01, and ***p < 0.001 (Student's t test, A: analysis of variance (ANOVA) with Bonferroni post-test).

Inducible regulatory T cells 35 (iTr35, *in vitro*) are a distinct subset of Tregs that are induced *in vitro* by treating human or mouse naive T lymphocytes with IL-35 22. iTr35 cells have strong immunosuppressive effects and exhibit long-term stability 22. However, the specific phenotypes and functions of Tr35 cells *in vivo* remain to be characterized. We previously generated IL-35 transgenic reporter mice using the BAC expression vector (35EbiT) 25. Here, we found that Foxp3-negative Tconvs marked by the reporter gene Thy1.1 (Ebi3) had high CD44 expression and low CD62L expression, indicating an activated state (Figure 1A-B). We sorted GFP^-^CD62L^low^Thy1.1^+^ and GFP^-^CD62L^hi^Thy1.1^-^ cells from double transgenic 35EbiT×Foxp3-KIGFP mice by flow cytometry and analyzed the mRNA expression levels of Ebi3, p35, and p28 by RT-PCR. The results showed that Thy1.1^+^ cells expressed high levels of Ebi3 and p35 mRNA but not p28 mRNA, confirming that these cells were Tr35 cells (Figure 1C). Further analysis by flow cytometry revealed that Tr35 cells had high expression of the activation-related molecules CD44, ICOS, CD38, and CD69, low expression of CD62L, and low expression of the anti-apoptotic protein Bcl2, indicating an activated state. Additionally, the expression of CTLA4, GITR, and PD1, molecules related to Treg function, was high in Tr35 cells and similar to that in Tregs. Overall, the phenotype of Tr35 cells was similar to that of IL-35-Tregs, a population of effector suppressor T cells (Figure 1D-E), suggesting that Tr35 cells have inhibitory activity *in vitro*. Our experimental results confirmed that Tr35 cells exhibited good inhibitory activity *in vitro*, further supporting their classification as Tregs (Figure S1).

### Functional analysis of Tr35 cell subsets *in vivo*

**Figure 2:**
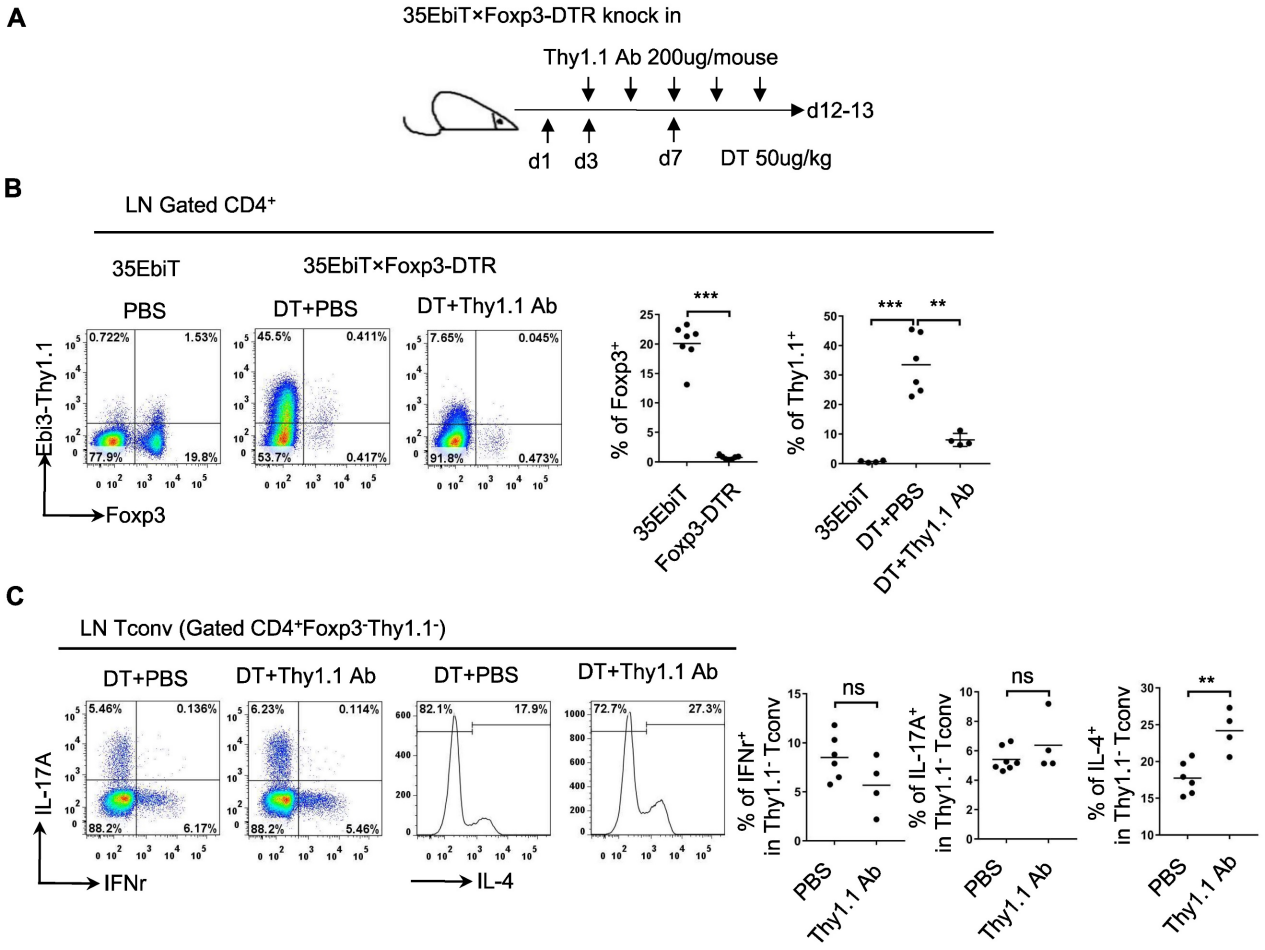
** Functional analysis of Tr35 cells *in vivo*. (A)** Schematic representation of the experimental procedure. Systemic deletion of Tregs was induced by administering 3 diphtheria toxin (DT) treatments to mice. During this period, Thy1.1 antibody treatments were administered 5 times at one-day intervals. **(B)** The flow plots represent the expression of Foxp3 and Thy1.1 (Ebi3) by CD4^+^ T cells at 12 days after the third DT treatment or PBS control. The average frequencies of Foxp3^+^ Treg cells and Tr35 cells among gated CD4^+^ T cells are shown. **(C)** Flow cytometry analysis of the expression of IL-17A, IFN-γ and IL-4 by CD4^+^Foxp3^-^Thy1.1^-^ Tconvs from the lymph nodes of DT- or PBS-treated 35EbiT-Foxp3-DTR mice. The average frequencies of cytokine-positive cells among gated subsets are shown. The data shown are typical results from two similar experiments, n=4-6. ns, not significant, **p < 0.01, and ***p < 0.001 (Student's t test).

Tregs can induce the production of Tr35 cells *in vitro* by secreting IL-35. To determine whether Tr35 cell production is dependent on Tregs *in vivo*, we generated 35EbiT-Foxp3-DTR double transgenic mice. In these mice, depletion of Foxp3-expressing cells can be induced by treatment with diphtheria toxin (DT) to study various immune disorders (Figure 2A) . After three DT treatments to induce systemic depletion of Tregs, less than 1% of CD4^+^ T cells expressed Foxp3 on day 12. Enlargement of the peripheral lymph nodes and spleen was observed at the end of the experiment (Figure S2A), and flow cytometry analysis showed that the absence of Tregs led to a significant increase (approximately 40-fold) in the generation of Tr35 cells (Figure 2B). These results suggested that Tr35 cell production can occur independently of Tregs. Flow cytometry analysis of relevant activation markers revealed that the Tr35 cells exhibited an activated state, with high expression of the activation molecules CD44, ICOS, CD69, and PD1, high expression of the cell proliferation marker Ki67, and low expression of the anti-apoptotic molecule Bcl2 (Figure S2B), consistent with the phenotype of steady-state mice (35EbiT). Further analysis of cytokine secretion revealed that systemic Treg depletion induced a strong Th2 inflammatory immune response marked by increased secretion of the Th2-related cytokine IL-4 (Figure S2C). Analysis of the relationship between IL-4 secretion and Tr35 cell generation showed that IL-4 secretion decreased with increased Thy1.1 (Ebi3) expression in CD4^+^ T cells (Figure S3A). Detection of Tr35 cells *in vivo* using Thy1.1 antibody analysis showed that Tr35 cells had a partial regulatory effect on the Th2 immune response but did not affect the secretion of IL-17A and IFN-γ by CD4^+^ T cells (Figure 2C) 27. *In vitro* induction of the directed differentiation of Th1, Th2, and Th17 cells by cytokines showed that more Tr35 cells were induced under Th2 polarization conditions (Figure S3B), suggesting that Tr35 cells are more involved in regulating the Th2 immune response.

### *In vivo* induction of Tr35 cells after multiple rounds of T-cell receptor stimulation

**Figure 3:**
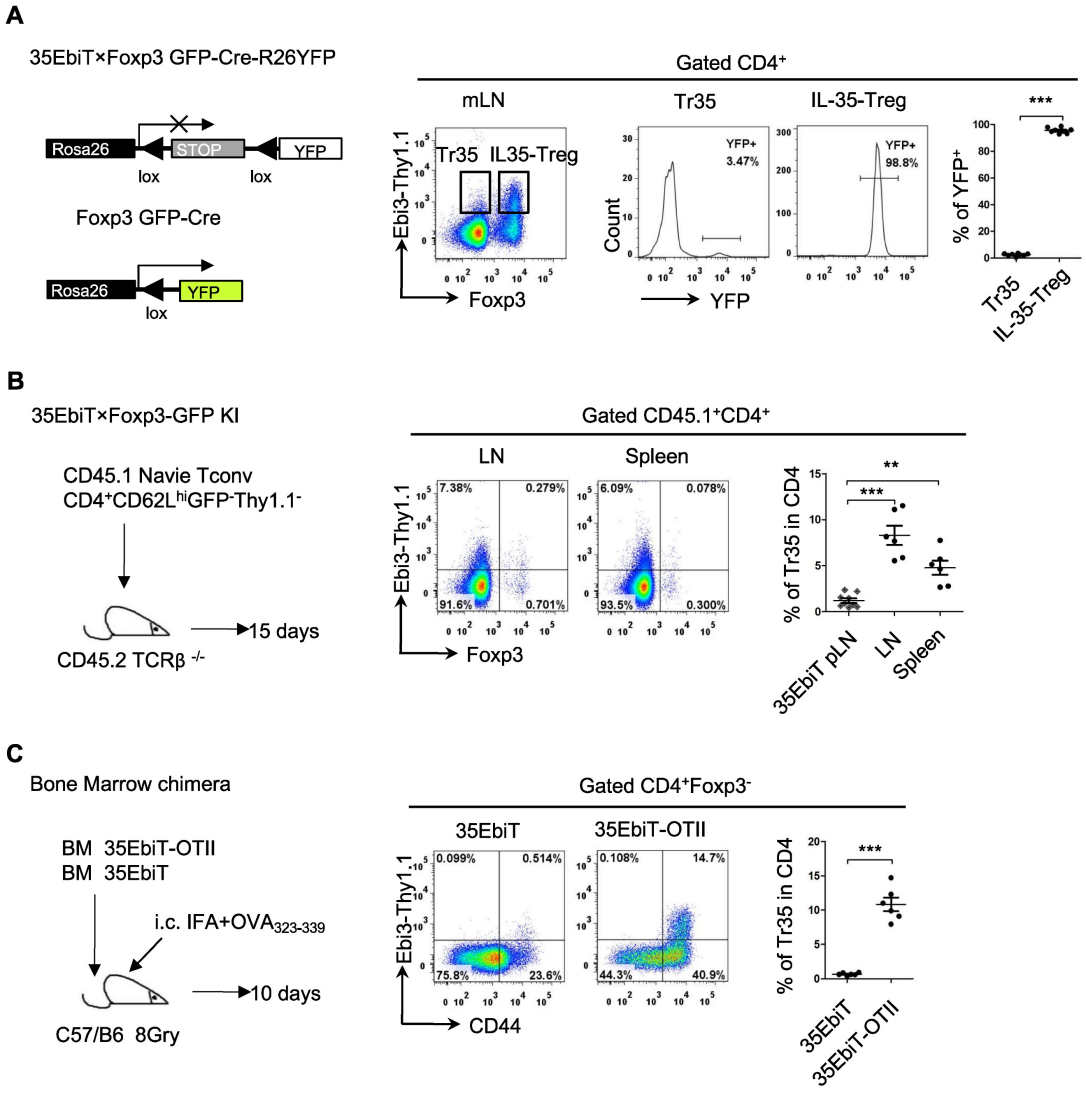
***In vivo* induction of Tr35 cells after multiple rounds of TCR stimulation. (A)** Flow cytometry analysis of cells from the mesenteric lymph nodes of 35EbiT×Foxp3GFP-Cre-R26YFP triple transgenic mice. The plot is gated on CD4^+^ T cells with secondary gating on specific subsets based on Foxp3 and Thy1.1 (Ebi3) expression levels. The numbers in the quadrants indicate the percentage of YFP^+^ cells in each gated subpopulation. The average frequencies of YFP^+^ cells in each gated subpopulation are shown. **(B)** FACS-purified CD45.1^+^CD4^+^GFP^-^CD62L^hi^Thy1.1^-^ fractions from 35EbiT×Foxp3-GFP KI mice were intravenously injected into sex-matched TCRβ KO recipient mice at 1 × 10^6^ cells/mouse (left). The flow plots represent the expression of Foxp3 and Thy1.1 (Ebi3) by donor CD45.1^+^CD4^+^ cells at 15 days after transfer. The average frequencies of Thy1.1 (Ebi3)^ +^cells in Foxp3^+^ or Foxp3^-^ subsets from the indicated organs of recipient mice are shown. **(C)** CD45.1^+^ 35EbiT-OTII and CD45.1^+^CD45.2^+^ 35EbiT bone marrow chimera mice were established and challenged with IFA+OVA^323-339^ peptide. The flow plots represent the expression of CD44 and Thy1.1 (Ebi3) by each gated subpopulation at 10 days after peptide challenge. The average frequencies of Thy1.1 (Ebi3)^ +^ cells in each gated subpopulation are shown. The data shown are typical results from three similar experiments, n=6. The error bars represent the mean ± SEM. **p < 0.01 and ***p < 0.001 (A, C: Student's t test, B: analysis of variance (ANOVA) with Bonferroni post-test).

Foxp3 expression is unstable in Tregs, and some Tregs lose Foxp3 expression and become exTregs 25, 26. Could the small number of Tr35 cells observed in mice actually be exTregs? To investigate the relationship between Tr35 cells and exTregs, we crossed 35EbiT mice with Foxp3creGFP×Rosa26-loxP-stop-loxP-YFP double-transgenic mice to generate triple-transgenic mice. In the Foxp3 fate-mapping system, exTregs are labeled with YFP. Analyzing the proportion of YFP^+^ cells (exTregs) within Tr35 cells (Foxp3^-^Thy1.1^+^) showed that only 3-4% of Tr35 cells were exTregs, indicating that the Tr35 cells were not mainly derived from exTregs (Figure 3A).

To further determine whether Tr35 cells differentiate and develop from naive CD4^+^ Tconvs, we adoptively transferred naive CD4^+^ Tconvs (CD4^+^GFP^-^CD62L^hi^Thy1.1^-^) into TCRβ knockout recipient mice. Flow cytometry results showed that the adoptively transferred naive CD4^+^ Tconvs could differentiate into Tr35 cells (Figure 3B), consistent with previous reports. These results indicated that Tr35 cells are a new type of suppressor T cell distinct from exTregs that are induced and differentiated from traditional CD4^+^ T cells *in vivo*.

Tr35 cells are highly activated, and it is not clear if they can be generated by T-cell receptor (TCR) signaling *in vivo*. To investigate this possibility, we constructed bone marrow chimeric mice by combining bone marrow cells from 35EbiT mice with bone marrow cells from 35EbiT-OTII mice at a ratio of 5:1. After transplantation, the mice were subcutaneously immunized with OVA polypeptide to activate 35EbiT-OTII cells. Flow cytometry analysis of the proportion of Thy1.1 (Ebi3) positive cells showed that the proportion of Tr35 cell subsets in OVA TCR-specific cells after immunization was significantly increased compared to non-TCR transgenic cell controls (Figure 3C). This indicated that activation of TCR signaling significantly increased the ability of CD4^+^ T cells to secrete IL-35.

### Tr35 and Tr1 cells are distinct populations of induced Tregs

**Figure 4:**
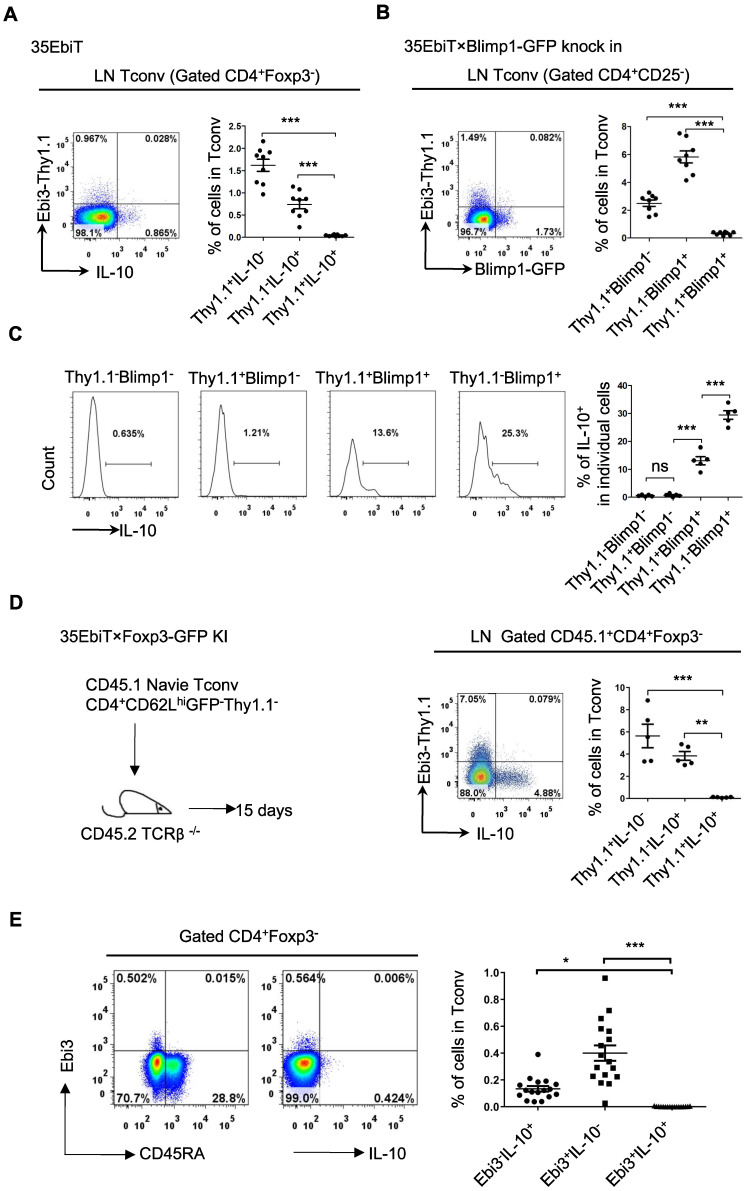
** Tr35 and Tr1 cells are distinct populations of Tregs. (A)** Plots of the expression of IL-10 and Thy1.1 (Ebi3) by CD4^+^Foxp3^-^ cells isolated from the lymph nodes of 35EbiT transgenic mice in the steady state. **(B)** Plots of the expression of GFP (Blimp1) and Thy1.1 (Ebi3) by CD4^+^Foxp3^-^ cells isolated from the lymph nodes of 35EbiT × Blimp1-GFP KI reporter mice in the steady state. **(C)** Flow cytometry analysis of the expression of IL-10 by each cell subset as indicated above the plots. The average frequency of IL-10^+^ cells in the gated subsets is shown. **(D)** FACS-purified CD45.1^+^CD4^+^GFP^-^CD62L^hi^Thy1.1^-^ fractions from 35EbiT×Foxp3-GFP KI mice were intravenously injected into sex-matched TCRβ KO recipient mice at 1 × 10^6^ cells/mouse (left). The flow plots represent the expression of IL-10 and Thy1.1 (Ebi3) by donor CD45.1^+^CD4^+^Foxp3^-^ cells at 15 days after transfer. **(E)** Plots of the expression of IL-10 and Thy1.1 (Ebi3) by CD4^+^ Tconvs (CD4^+^Foxp3^-^) from human PBMCs. The data shown are typical results from three similar experiments, n=5-9 (A-D), n=17 (E). The error bars represent the mean ± SEM. ns, not significant, *p < 0.05, **p < 0.01, and ***p < 0.001 (analysis of variance (ANOVA) with Bonferroni post-test).

The inhibitory cytokines IL-35 and IL-10 share many properties and are mainly secreted by effector Tregs, which can induce Tconvs to transform into inhibitory T cells (Tr35 and Tr1) both *in vitro* and *in vivo*, even if neither the Tr35 nor the Tr1 express Foxp3 22,28. The relationship between the subsets of labeled Tr35 and Tr1 cells in mice was investigated by isolating mesenteric lymph node cells, stimulating cytokine expression *in vitro*, and determining the proportions of IL-35-secreting Tr35 and IL-10-secreting Tr1 cells by flow cytometry. The results showed that Tr35 cells and Tr1 cells were different cell subsets, with very few cells producing both cytokines simultaneously (Figure 4A).

Blimp1 is a key transcription factor that regulates IL-10 expression. To determine whether Blimp1 is also a key transcription factor for IL-35 secretion by Tr35 cells, 35EbiT×Blimp1-GFP double transgenic mice were bred. Tr35 cells isolated from these mice did not express the Blimp1 molecule, which suggested that IL-35 secretion by Tr35 cells does not depend on Blimp1, consistent with our previous findings in Tregs (Figure 4B). By contrast, the Blimp1-positive cells were Tr1 cells that secreted IL-10. Thy1.1^+^Blimp1^+^ double-positive cells were detectable but at a significantly lower frequency, and they also produced IL-10 but at much lower levels than Thy1.1^-^Blimp1^+^ cells (Figure 4C).

To further investigate the differentiation and development of Tr35 and Tr1 cells *in vivo*, naive CD4^+^ Tconvs were adoptively transferred to TCRβ knockout recipient mice. Flow cytometry results showed that Tr35 and Tr1 cells were all induced and differentiated from traditional CD4^+^ T cells *in vivo*. However, the Tr35 and Tr1 cells formed distinct subpopulations (Figure 4D). Finally, human peripheral blood mononuclear cells (PBMCs) were stimulated *in vitro*, and the proportions of IL-35- and IL-10-secreting Tconvs were determined by flow cytometry. The results showed that human Tr35 cells were in an activated state with low expression of CD45RA and were a different cell subset from human Tr1 cells (Figure 4E). Overall, the results for both mouse and human specimens indicate that Tr35 and Tr1 cells are independent inducible suppressor cell subsets.

### Tr35 and Tr1 cells have different activation stages and distinct expression patterns

**Figure 5:**
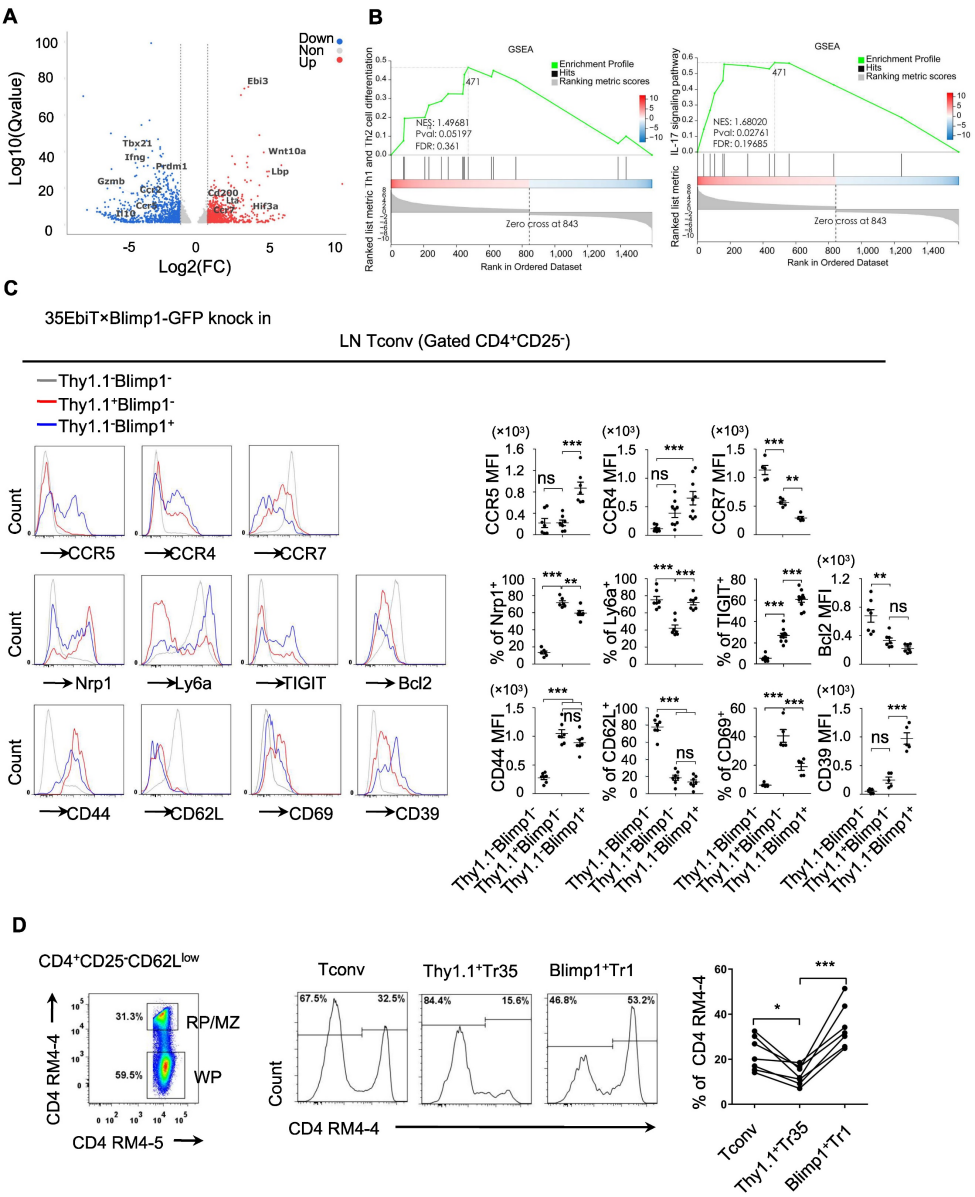
** Tr35 and Tr1 cells have different activation stages and tissue distributions. (A)** Tr35 (CD4^+^CD25^-^CD62L^low^Thy1.1^+^) and Tconv (CD4^+^CD25^-^CD62L^-^Thy1.1^-^) fractions from 35EbiT × Foxp3-DTR KI reporter mice were purified by FACS and then used for RNA-seq analysis. The volcano plot of selected genes (log_2_-fold change) that were differentially expressed (>1.0-fold) between Tr35 cells and Tconvs is shown for quadruplicate samples. The colors indicate upregulated (red) and downregulated (blue) genes in the Tr35 subset compared to the Tconv subset. **(B)** Enrichment plots from the gene set enrichment analysis (GSEA). Th1 and Th2 or Th17 cell differentiation pathways were enriched in differentially expressed genes (Tr35 versus Tconv). **(C)** Flow cytometry analysis of CD4^+^ T cells isolated from the lymph nodes of 35EbiT × Blimp1-GFP KI reporter mice in the steady state. The histogram plots are gated on Thy1.1^-^Blimp1^-^ (gray line), Thy1^+^Blimp1^-^ (red line), and Thy1.1^-^Blimp1^+^ (blue line) cells (left). The percentages and MFI of the indicated markers are shown (right). **(D)** *In vivo* CD4 labeling. The representative flow cytometry plot depicts the dual-labeled RP/MZ and single-labeled WP cells. The histograms (middle panel) and graph (right) show the frequencies of Tconvs (Thy1.1^-^Blimp1^-^), Tr35 cells (Thy1.1^+^Blimp1^-^), and Tr1 cells (Thy1.1^-^Blimp1^+^) labeled *in vivo* with CD4-biotin. The data shown are typical results from three similar experiments, n=3 (A-B), n=5-7 (C-D). The lines connecting the points represent statistical analyses comparing different cell populations within the same mouse. The error bars represent the mean ± SEM. ns, not significant, *p < 0.05, **p < 0.01, and ***p < 0.001 (analysis of variance (ANOVA) with Bonferroni post-test).

To gain a deeper understanding of what distinguishes the Tr35 and Tr1 cell populations, we compared the transcriptomes of the Tconv and Tr35 subsets by performing RNA-seq analysis. We identified 763 upregulated genes and 890 downregulated genes in Tr35 cells compared to Tconv cells (Figure 5A). The upregulated genes included Ebi3, Lta, and CD200, whereas the downregulated genes included Prdm1, Gzmb, and IL-10 (Table S1). Further pathway analysis using genome set enrichment analysis (GSEA) revealed that the Tr35 genes were enriched in Th1, Th2 and Th17 signaling pathways, suggesting that Tr35 cells may downregulate the differentiation of Th1, Th2 and Th17 subgroups (Figure 5B). Comparing the gene expression profiles of Tr35 cells, Tconvs, and previously described Tr1 cells revealed that Tr35 cells had a unique gene expression pattern distinct from those of Tconvs and Tr1 cells (Figure S4A) 29. Gene clustering analysis also revealed that the gene expression trend of Tr35 cells differed from that of Tr1 cells (Figure S4B). Among transcription factor-related genes, Tr1 cells had higher expression of *Prdm1* and *Junb* 30,31, whereas Tr35 cells had higher expression of *Hif3a* and *Ezh2* (Figure S4C). With respect to genes related to secretion or cytokines, Tr35 cells had higher expression of Ebi3, IL-2, and Lta and lower expression of the inflammatory cytokines IL-17A and IFN-γ (Figure S4D). By contrast, Tr1 cells 32,33 had higher expression of IFN-γ and immune regulatory molecules such as Gzmb, Gzmk, Prf1, and IL-10. Among chemotaxis-related genes, Tr1 cells had higher expression of chemotaxis-related molecules such as CCR5, CCR9, and ICAM1, whereas Tr35 cells had higher expression of CCR7 (Figure S4E), the main chemokine receptor that guides T-cell localization into the T-zone in secondary lymphoid organs. We confirmed high expression of cytokines IL-2 and TNF-α in Tr35 cells by flow cytometry (Figure S4F).

We confirmed the differential expression of various genes by flow cytometry of cells from 35EbiT-Blimp1-GFP gene reporter mice, which allowed us to analyze the differentially expressed molecules between Tr35 and Tr1 cell subsets and preliminarily determine whether their migration and tissue localization differed. The flow cytometry results showed that both Tr35 and Tr1 cells had characteristics of activated T cells, including high expression of CD44 and low expression of CD62L and the anti-apoptotic protein Bcl2 compared with double-negative (Blimp1^-^Thy1.1^-^) CD4^+^ T cells. Tr35 cells specifically expressed high levels of the CD69 molecule and low levels of Ly6a, whereas Tr1 cells expressed high levels of CD39, TIGIT, Ly6a and low levels of CD69 (Figure 5C).

Analyzing the expression of chemokine receptors on the cell surface revealed that Tr1 cells expressed higher levels of CCR5, CCR4, and other chemokine receptors and lower levels of CCR7 (Figure 5C), suggesting that Tr1 cells are more likely to migrate out of lymphoid organs to the periphery to exert inhibitory functions. However, Tr35 cells had low expression of CCR5 and CCR4 and high expression of CCR7 (Figure 5C), suggesting that Tr35 cells are not easily able to migrate to peripheral organs and mainly remain in the lymphoid organs, where they can exert their regulatory function. To test this hypothesis, we injected mice with a CD4 antibody (RM4-4) via the tail vein to label CD4^+^ T cells distributed in the red pulp and MZ regions of the spleen *in vivo*, and then used flow cytometry to collect and analyze the total activated CD4^+^CD25^-^CD62L^low^ T cells. We divided the total activated T cells into three groups according to the expression of Thy1.1 and Blimp1: Tr35 (Thy1.1^+^Blimp1^-^), Tr1 (Thy1.1^-^Blimp1^+^), and eTconv (Thy1.1^-^Blimp1^-^). Cell migration status was analyzed by counting the proportion of CD4-labeled T cells among the three populations of cells. The results showed that in the red pulp regions, the proportion of labeled Tr35 cells was approximately 15%, whereas the proportion of labeled Tr1 cells was much higher (about 30%) (Figure 5D). This result indicated that the two groups of Tr35 and Tr1 cells had different tissue localizations in the spleen, consistent with our hypothesis.

### Genetic tracing of Tr35 regulatory cell subsets

**Figure 6:**
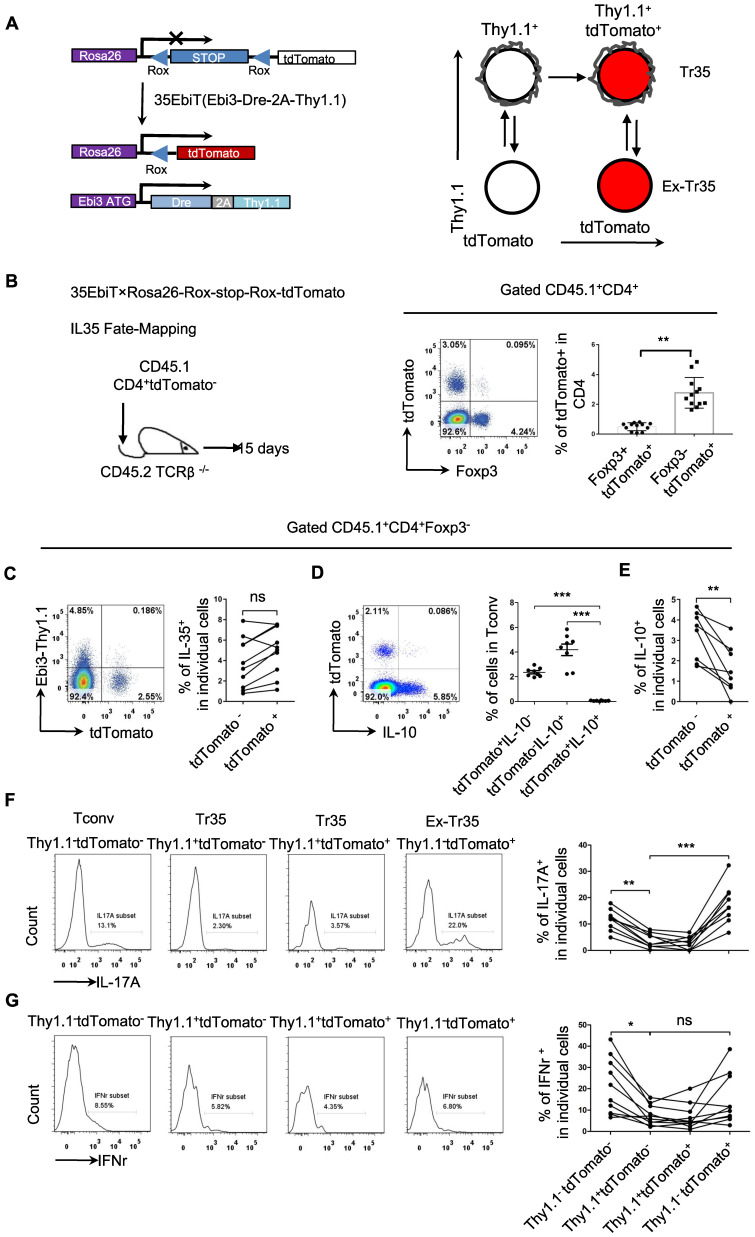
** Genetic fate mapping of Ebi3 expression in Tr35 cells. (A)** Schematic map for using dual transgenic Ebi3 reporter mice to trace Ebi3-expressing cells and their progeny. **(B)** FACS-purified CD45.1^+^CD4^+^tdTomato^-^ fractions from 35EbiT×Rosa26-Rox-stop-Rox-tdTomato mice were intravenously injected into sex-matched TCRβ KO recipient mice at 1 × 10^6^ cells/mouse (left). The flow plots represent the expression of tdTomato by donor CD45.1^+^CD4^+^ cells at 15 days after transfer. **(C)** Flow cytometry analysis of the expression of Thy1.1 and tdTomato by CD4^+^ Tconvs from the lymph nodes of recipient mice. The numbers in quadrants indicate the percentage of cells in each. **(D)** Flow cytometry analysis of the expression of IL-10 and tdTomato by CD4^+^ Tconvs from the lymph nodes of recipient mice. The numbers in quadrants indicate the percentage of cells in each. **(E)** Average frequency of IL-10 expression in tdTomato^+^ cells from the lymph nodes of recipient mice. **(F)** Flow cytometry analysis of the expression of IL-17A by each cell subset as indicated above the plots. The average frequency of IL-17A^+^ cells in the gated subsets is shown. **(G)** Flow cytometry analysis of the expression of IFN-γ by each cell subset as indicated above the plots. The average frequency of IFN-γ^ +^ cells in the gated subsets is shown. The data shown are typical results from three similar experiments, n=8-12. The lines connecting the points represent statistical analyses comparing different cell populations within the same mouse. The error bars represent the mean ± SEM. ns, not significant, *p < 0.05, **p < 0.01, and ***p < 0.001 (B,C,E: Student's t test, D,F,G: analysis of variance (ANOVA) with Bonferroni post-test).

Analyzing the expression of chemokine receptors on the surface of Tr35 and Tr1 cells revealed that these cells had different activation statuses, which raises the question of whether they represent two stages of T-cell differentiation or two distinct populations. To clarify the developmental relationship between Tr35 and Tr1 cells, we conducted genetic fate mapping by crossing 35EbiT mice with the Rosa26-rox-tdTomato tracer strain. In the resultant mice, Dre expression in Thy1.1 (Ebi3)^+^ T cells allows the excision of the Rox-flanked Rluc-stop cassette and drives constitutive transcription of tdTomato, permanently labeling Thy1.1 (Ebi3)^+^ T cells and their progeny (Figure 6A). To exclude the influence of tdTomato expressed by hematopoietic stem cells, we adoptively transferred peripheral mature CD4^+^tdTomato^-^ T cells (sorted from pooled lymph nodes and spleens of 35EbiT-Rosa26-rox-tdTomato reporter mice) intravenously into TCRβ^-/-^ recipients (Figure 6B). In this setting, only mature CD4^+^ T cells can turn on tdTomato expression and mark Ebi3 gene transcription. Fifteen days after transfer, we detected a clear population of T cells labeled by tdTomato (Figure 6B). Interestingly, the tdTomato-labeled T cells contained far more Foxp3-negative Tconvs than Foxp3-positive Tregs (Figure 6B), unlike the results of real-time labeling of Ebi3-expressing cells with Thy1.1 (Ebi3) 25. Based on the analysis of Thy1.1 (Ebi3) expression in tdTomato^+^ cells, it was observed that Tr35 cells exhibited significant transdifferentiation. Only a small fraction of tdTomato^+^ cells (<10%) retained the Thy1.1 (Ebi3) expression observed in donor CD4^+^ Tconvs (Figure 6C). In summary, the Tr35 cells displayed a higher degree of transdifferentiation compared to IL-35-Tregs 34, with the majority of tdTomato-labeled Tconvs representing cells that had previously expressed IL-35. We obtained Tr35 and IL-35-Treg cells through sorting and transferred them separately to mice irradiated with 5Gry. Even on day 21, transferred cells were still detectable. Examination of Thy1.1 (Ebi3) expression revealed that only around 10% of Tr35 cells were maintained IL-35 expression, while IL-35-Treg cells still had a higher proportion of cells expressing IL-35 (Figure S5).

Although the genetically traced Thy1.1 (Ebi3)^+^ cells exhibited transdifferentiation, the tdTomato^+^ cells and IL-10-producing Tr1 cells formed distinct subpopulations (Figure 6D). Further analysis of IL-10 expression revealed that tdTomato^+^ cells secreted significantly less IL-10 than tdTomato^-^ cells, supporting the notion that Tr35 cells represent a general intermediate activation stage of effector T-cell differentiation (Figure 6E). As a crucial type of helper T cell, CD4^+^ cells can differentiate into various function-specific cell subsets and secrete corresponding inflammatory cytokines. Based on the expression of Thy1.1 (Ebi3) and tdTomato, the CD4^+^ cells were divided into three subsets: Tconv (Thy1.1^-^tdTomato^-^), Tr35 (Thy1.1^+^tdTomato^-^ or Thy1.1^+^tdTomato^+^), and exTr35 (Thy1.1^-^tdTomato^+^). We performed a statistical analysis of the expression of the inflammatory cytokines IL-17A (Figure 6F) and IFN-γ (Figure 6G) in these three groups of cells. Tr35 cells had a significantly lower ability to secrete IL-17A and IFN-γ than the other cell types. By contrast, the ability of exTr35 cells, which had lost Thy1.1 (Ebi3) expression, to secrete inflammatory cytokines was restored, indicating that the inhibitory cytokine IL-35 induces the production of Tr35 cells *in vivo* and simultaneously inhibits the expression of inflammatory cytokines.

### Tr35 cells prevent OVA-induced allergic airway inflammation

Allergic asthma is a common respiratory disease characterized by airway inflammation, hyperresponsiveness, and mucus hypersecretion. Tregs, a subset of CD4^+^ T cells, play a crucial role in maintaining immune homeostasis and suppressing pathological immune responses. However, the therapeutic potential of antigen-specific Tr35 cells, a subset of induced Tregs that produce IL-35, in allergic asthma has not been established.

**Figure 7:**
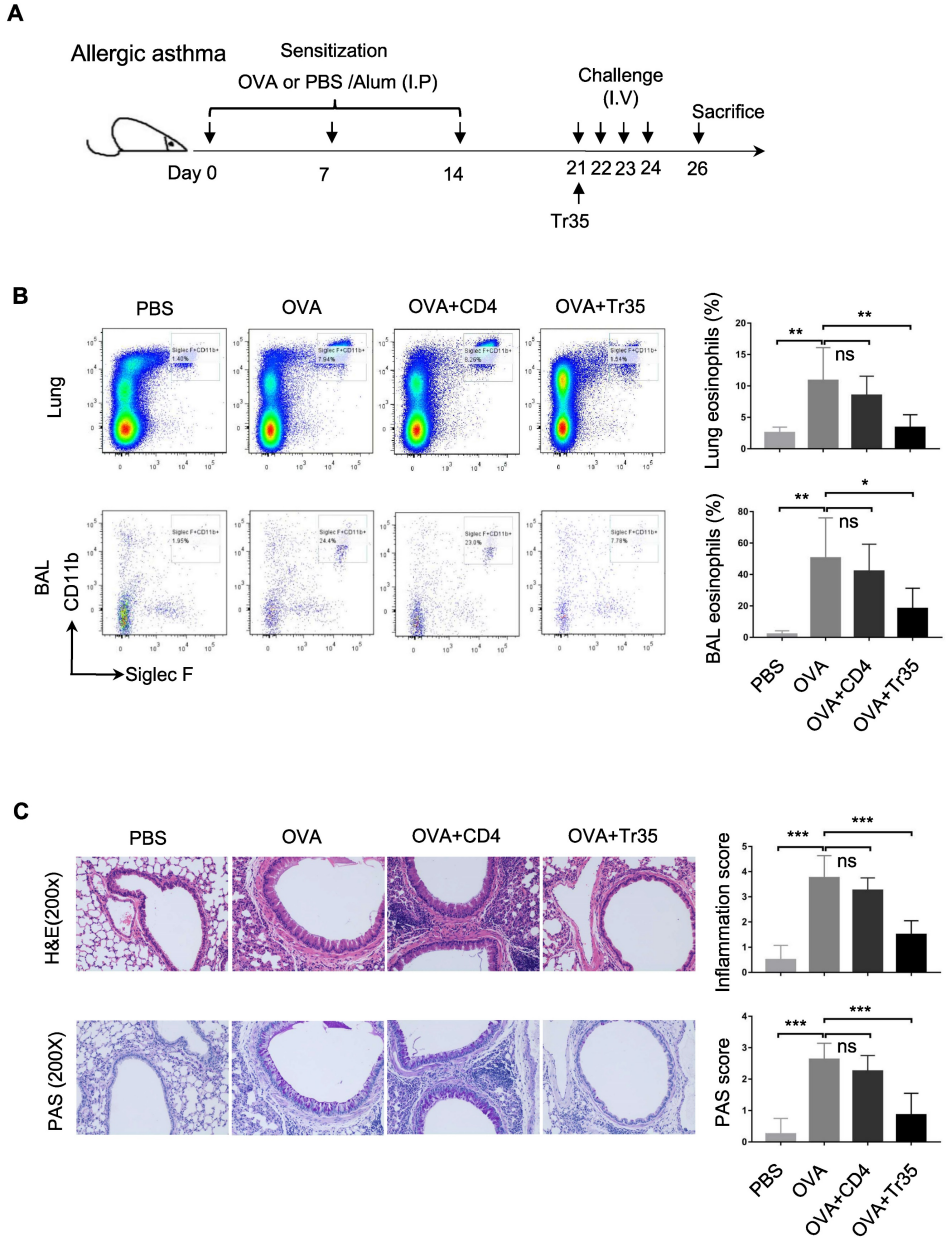
** OVA-specific Tr35 cells prevent OVA-induced allergic airway inflammation. (A)** Schematic representation of the experimental procedure. Mice were sensitized with three intraperitoneal injections of ovalbumin (OVA) or PBS in an alum adjuvant at days 0, 7 and 14. The mice received 2 × 10^5^ OVA-specific Tr35 cells (OT-II) by intravenous adoptive transfer after the first 1% OVA aerosol challenge on day 21. The mice were challenged daily for the next four days and sacrificed 48 h after the last aerosol challenge. **(B)** The percentages and numbers of eosinophils (Siglec F^+^CD11b^+^CD11c^-^) in the lungs and bronchoalveolar lavage (BAL) fluid were assessed by flow cytometry. **(C)** Lung sections were stained with HE and PAS to measure the numbers of infiltrated inflammatory cells and mucus-secreting cells. (200x). The data shown are typical results from three similar experiments, n=4-7. The error bars represent the mean ± SEM. ns, not significant, *p < 0.05, **p < 0.01, and ***p < 0.001 (analysis of variance (ANOVA) with Bonferroni post-test).

We investigated the therapeutic effects of OVA-specific Tr35 cells (35EbiT-OTII) in an OVA-induced allergic airway disease model. First, we adoptively transferred 2 × 10^5^ OVA-specific Tr35 cells into OVA-sensitized mice intravenously after an initial challenge with 1% OVA (Figure 7A). We then analyzed the efficacy of Tr35 cell therapy by assessing the proportions of eosinophils in lung tissue and bronchoalveolar lavage fluid by flow cytometry analysis (Figure 7B) and by histopathological examination of lung tissues using hematoxylin and eosin (HE) and PAS staining (Figure 7C). Compared with untreated mice, mice treated with Tr35 cells exhibited significant reductions in the proportions of eosinophils in lung tissue and bronchoalveolar lavage fluid, inflammatory cell infiltration in the lungs, and mucus hypersecretion. These findings suggest that OVA-specific Tr35 cells have the potential to regulate airway inflammation and alleviate OVA-induced allergic asthma.

## Discussion

In this study, we described the functions and characteristics of a new subset of Tregs, Tr35 cells, which do not express Foxp3 but secrete IL-35. Our study adds valuable insights to the existing knowledge about Tr35 cells. Using a novel IL-35 reporter mouse, we were able to distinguish IL-35-expressing cells based on the surface expression of Thy1.1 (Ebi3). Our findings reveal that Tr35 cells express high levels of molecules related to T-cell activation and can inhibit T-cell proliferation both *in vitro* and* in vivo*. Importantly, our results demonstrate that Tr35 cells are a distinct subpopulation of Tregs that are independent of Tr1 cells, with a unique gene expression profile and tissue distribution. The differentiation and developmental trajectories of Tr35 cells were tracked and analyzed using double transgenic reporter mice. The proportion of cells transiently expressing Thy1.1 (Ebi3) was higher in Tr35 cells than in IL-35-Treg cells *in vivo*, indicating that Tr35 cells regulate the expression of IL-35 more rapidly than IL-35-Treg cells.

Moreover, our data suggest that Tr35 cells play a critical role in regulating inflammatory immune responses by downregulating the expression of the inflammatory cytokines IFN-γ and IL-17A. Tr35 cells reinfusion significantly reduced airway hyperresponsiveness and lung inflammation in the OVA-induced allergic airway disease mouse model. Therefore, our findings suggest that Tr35 cells have therapeutic potential for inflammatory diseases.

Secreting suppressive cytokines is an important mechanism by which the immune system regulates immune responses and maintains regional immune tolerance 28,35. Clear inhibitory functions of TGFβ, IL-10, and IL-35 have been established. These cytokines are mainly secreted by Tregs and inhibit the function of effector immune cells by interacting with cytokine receptors. At the same time, they can induce the CD4^+^ T cells to differentiate into Tregs that secrete the same suppressive cytokines according to a pathway known as infectious tolerance. IL-35 is a newly discovered suppressive cytokine that has been shown to have strong inhibitory activity both *in vivo* and *in vitro* 22. CD4^+^ Tconvs pretreated with IL-35 *in vitro* can differentiate into a new type of suppressive T cell called iTr35 cells 22. Most research on these cells is based on *in vitro* induction of iTr35 cells after multiple rounds of stimulation, and their phenotype and functions may not reflect the actual *in vivo* state. We previously generated a novel IL-35 reporter mouse (Ebi3-Dre-Thy1.1, 35EbiT) that allows Ebi3-expressing cells to be distinguished by the surface expression of Thy1.1. In 35EbiT transgenic mice, CD4^+^Foxp3^-^ cells that secrete IL-35 can be detected by Thy1.1 antibody staining, and we refer to these cells as Tr35 cells (iTr35 cells *in vitro*). Tr35 cells are a group of induced suppressive T cells that exist under normal physiological conditions *in vivo* and do not require any induction treatment *in vitro*. Therefore, using the 35EbiT transgenic mice we could study the true phenotype and function of Tr35 cells *in vivo* state.

The regulation of IL-35-producing cells at the molecular level is not fully understood. Although the proportion of Tregs expressing IL-35 was higher than the proportion of Tr35 cells, the proportion of exTr35 cells that once expressed IL-35 was much higher than the proportion of Tregs that once expressed IL-35. In our previous study of IL-35-Tregs, we found that Foxp3 is not required for IL-35 expression; however, mutations in Foxp3 significantly reduce IL-35 expression in Tregs 25. We speculate that Foxp3 mutations affect the stability of IL-35 expression in Tregs to more closely resemble Tr35 cells. The Foxp3 gene itself or its regulatory Treg signature genes may be key molecules for stable IL-35 expression in Tregs and Tr35 cells 7,36. Epigenetic modifiers also contribute to the regulation of IL-35 expression. For instance, DNA methylation and histone acetylation both can modulate IL-35 expression in immune cells 37,38. These findings highlight the importance of epigenetic regulation in controlling IL-35 expression and immune responses. Other mechanisms are also involved in the regulation of IL-35 expression. cGAMP induces IL-35 expression by B cells through an IRF3-dependent pathway that is independent of type I interferon 39. Additionally, IL-35 expression is upregulated via JNK/c-Jun signaling mediated by HBx 40. This pathway leads to the transactivation of the IL-35 subunits Ebi3 and p35 promoter, thereby promoting IL-35 production. In a mouse model of OVA-induced allergic airway disease, IL-35 reduces the symptoms of the disease by inhibiting the recruitment of inflammatory dendritic cells (DCs) at the site of inflammation and draining lymph nodes 41. IL-35 therapy effectively treats allergic airway inflammation caused by memory/effector Th2 cells that react to allergen mites 42. Although we found that IL-35 expression is transient in Tr35 cells, Tr35 cell reinfusion therapy resulted in good inhibition of the progression of allergic airway disease. Future research should investigate the epigenetic regulation of the IL-35 (Ebi3) locus, which is crucial for the timely expression of IL-35. Understanding the mechanism of stable IL-35 expression will facilitate the development of cell products with better immunomodulatory functions and enhance the therapeutic value of Tr35 cell infusion.

We did not conduct a detailed comparative study of the immune regulatory functions of Tr35 and Tr1 cells. Instead, we focused on the immune regulatory function of Tr35 cells. In our 35EbiT-Foxp3-DTR double transgenic mouse model, the immune response induced by the deletion of Tregs was mainly Th2 type 43. When Tr35 cells were simultaneously deleted, the ability of CD4^+^ T cells to secrete IL-4 increased significantly. In addition, Th2-type polarization conditions induced the highest proportion of Tr35 cells *in vitro*, suggesting that the immune regulatory function of Tr35 cells is more closely related to Th2-type inflammatory immunity. Moreover, in the 35EbiT genetic fate-mapping transfer model, Tr35 cells significantly downregulated their own expression of the inflammatory cytokines IFN-γ and IL-17. Once Tr35 cells stopped expressing IL-35, their expression of inflammatory cytokines recovered, suggesting regulatory effects of Tr35 cells on Th1 and Th17 immune responses 44. The decrease in IL-4 secretion and increased Thy1.1 (Ebi3) expression during Tconv or Th2 cell conversion to Tr35 cells suggests a regulatory role of IL-35. IL-35 may act as a negative feedback mechanism, downregulating IL-4 to dampen the Th2 immune response 27. This phenotypic shift towards Tr35 cells could be driven by the Th2 microenvironment. Understanding these mechanisms could have implications for treating Th2-mediated inflammation. We speculate that Tr35 cells can regulate multiple types of inflammatory immune responses, depending on the immune microenvironment. Further in-depth research is needed to determine the specific immune regulatory mechanism.

IL-35 was initially identified as an inhibitory cytokine derived from Tregs 5, and its receptor was first discovered in T cells. The IL-35 receptor is an IL-12Rβ2/gp130 heterodimer or a homodimer of one chain 36. Whereas gp130 is constitutively expressed in various cell types, IL-12Rβ2 is primarily expressed by activated T cells, natural killer cells, B cells, and dendritic cells 45. Theoretically, cells expressing the IL-12Rβ2 receptor can receive stimulatory signals from IL-35 and be induced into suppressive cell types that secrete IL-35. Recent studies have significantly expanded the list of IL-35-producing cells to encompass various immune cell types. Regulatory B cells (Bregs) have been identified as a significant source of IL-35 production 46. These Bregs play a crucial role in immune regulation during autoimmune and infectious diseases 7. These specialized regulatory cells contribute to immune tolerance and suppression of immune responses. In addition to Bregs, Tregs, and tolDCs 47,48, other cell types have also been found to produce IL-35. CD4^+^ conventional T cells (Tconvs) have been shown to produce IL-35, contributing to the regulation of immune responses 22. Tumor-associated macrophages, which are involved in tumor progression and immune evasion, have been identified as a source of IL-35 production 49. Although our study specifically focused on the secretion of IL-35 by CD4^+^ T cells, in 35EbiT-Rosa26-rox-tdTomato reporter mice, we observed tdTomato expression in some B cells and CD8^+^ T cells, suggesting that multiple cell types can secrete IL-35, at least in mice. The characteristics of tdTomato expression cells and their mechanisms regulating the expression of cytokines require further investigation. In addition, the IL-12 family of cytokines is composed of heterodimers formed by α and β chains 35. Does the Ebi3 binding repertoire extend beyond p28 and p35? A recent study described a novel interaction between Ebi3 and p19 that induces the expression of IL-39 in activated B cells 50,51. It is important to carefully discern cytokine types when using 35EbiT transgenic mice for research.

Our findings have several important implications. First, our results highlight the importance of studying immune cell function *in vivo*, as we were able to demonstrate that Tr35 cells regulate the expression of IL-35 more rapidly *in vivo* than *in vitro*. In our study, we employed specific double transgenic reporter mice to investigate the *in vivo* characteristics of Tr35 cells. The models utilized allowed us to trace the fate of Tr35 cells and analyze their behavior in living organisms. Second, our data suggest that there are multiple pathways of Treg development, and further research is needed to fully understand the heterogeneity of IL-35 expression cells. Third, our findings indicate that Tr35 cells have a unique role in regulating immune responses by modulating the secretion of inflammatory cytokines in real time through the regulation of IL-35 expression. Finally, our findings could have important implications for the development of new therapies for inflammatory diseases in humans, and further research is needed to investigate the safety and efficacy of Tr35 cell-based therapies. Our findings provide a promising starting point for further investigation of the molecular mechanisms underlying the function of Tr35 cells.

## Materials and methods

### Mice

35EbiT and Rosa26-Rox-tdTomato reporter mice were described previously 25. C57BL/6 (B6) and CD45.1^+^ B6 congenic mice were purchased from the Peking University laboratory animal center (Beijing, China). Foxp3GFP-Cre and R26YFP reporter mice were described previously 26. Foxp3-DTR KI mice were purchased from Jackson Lab (Stock No: 016958). Foxp3-GFP KI mice were a gift from Fuping Zhang (Institute of Microbiology, CAS). OT-II (Y-linked) mice and Blimp1-GFP KI mice were provided by Baidong Hou (Institute of Biophysics, CAS). All mice were housed in a specific pathogen-free animal facility in accordance with the guidelines for the care and use of laboratory animals established by the Beijing Association for Laboratory Animal Science. All mouse experiments were performed in accordance with the regulations of the Research Ethics Committee of the Institute of Microbiology, Chinese Academy of Sciences. The protocol was approved by the Research Ethics Committee of the Institute of Microbiology, Chinese Academy of Sciences (permit number APIMCAS2021069).

### Construction of the bone marrow chimera mouse model

The donor mice were euthanized and immersed in 75% ethanol for disinfection. The femur and tibia bones were isolated and the muscles were removed using Kimwipes. The bones were then immersed in 75% ethanol for 2 min and washed twice with PBS. The ends of the leg bones were cut off using bone shears, and the bone marrow inside the bones was flushed out using a syringe with a 27G (0.4 mm) needle. The bone marrow single-cell suspension was obtained by filtering through a 40 μm nylon mesh. After cell counting, the required number of bone marrow cells were centrifuged at 1200 rpm for 5 min and resuspended in pre-chilled sterile PBS. Then the bone marrow cells were mixed according to experimental requirements from different donor mice. The recipient mice were irradiated with a lethal dose (8Gy, one time) using a γ-irradiator to eliminate the hematopoietic cells. Within 24 hours, the donor bone marrow cells (at least 1x10^6^ per mouse) were transferred to the irradiated recipient mice through tail vein injection. Antibiotics were added to the drinking water of the mice for 2 weeks to prevent mouse mortality due to secondary infections. After 8 weeks of reconstitution, the mice were immunized subcutaneously by injecting a mixture of 50 μL of OVA323-339 peptide (1 mg/mL) and adjuvant around the inguinal lymph nodes. Ten days later, the lymph nodes were harvested for immune analysis.

### Antibodies

Fluorescently labeled anti-Thy1.1 (OX-7), anti-CD25 (PC61), labeled anti-CD4 (GK1.5), anti-CD8 (53-6.7), anti-CD44 (IM7), anti-CD69 (H1.2F3), anti-CD38 (90), anti-CD152 (UC10-4B9), anti-CD357 (DTA-1), anti-IL-10 (JES5-16E3), anti-CD278 (17G9), anti-CD279 (RMP1-30), anti-CD103 (2E7), anti-CD62L (MEL14), anti-CCR7 (4B12), anti-Ly-6a/E(D7), anti-Foxp3 (FJK-16s), anti-IFN-γ (XMG1.2), anti-IL-17A (eBio17B7), anti-CD4 biotin (RM4-4), anti-CD4 (RM4-5), anti-CD45.1 (A20), anti-CD45.2 (104), anti-Bcl-2 (BCL/10C4), CD16/32(2.4G2), and eFluor®506 fixed viability dye were purchased from BD Pharmingen, BioLegend or eBioscience. Biotinylated neuropilin-1 pAb (BAF566) was purchased from R&D Systems.

### Cell isolation and flow cytometry

Single-cell suspensions were prepared from the spleens and lymph nodes of various strains of mice by mechanical disruption in DMEM supplemented with 2% (vol/vol) FCS, penicillin (100 IU/ml), and streptomycin (100 IU/ml). Splenocytes were treated with 1 × ACK to lyse red blood cells. Antibody mixture staining was performed in ice-cold buffer (DMEM with 2% FCS) for 30 min with dilutions of antibodies. Stained single-cell suspensions were analyzed using an LSRFortessa flow cytometer running FACSDiva software (BD Biosciences). FSC 3.0 files were analyzed and presented using FlowJo software (TreeStar).

### PBMCs isolation

Blood samples were mixed with phosphate buffered saline (PBS) at a 1:1 ratio. Peripheral blood mononuclear cells (PBMCs) were isolated using Ficoll-Hypaque density 1.077 g/ml (Sigma Aldrich). Briefly, 5 mL of diluted blood was layered onto Ficoll-Hypaque in a tube and centrifuged for 20 min at 400 g. The cells in the middle layer were collected and washed three times. The supernatant was removed, and the PBMCs were collected as a pellet. The cells were then resuspended in PBS to a concentration of 1x10^7^ cells/ml for further experiments.

### Cytokine analysis

PBMCs or Lymphocytes cells were incubated with 0.5 mM ionomycin, 10 ng/ml PMA, and 3 mM monensin at 37°C and 5% CO_2_ for 3-4 h. After surface antibody staining, the cells were fixed and permeabilized using the Foxp3 Fixation/Permeabilization kit (eBioscience, 00-5523-00) and stained to detect intracellular proteins. To stain Foxp3 with GFP, tdTomato, and YFP, the cells were fixed in 1% paraformaldehyde (PFA) for 4 min at room temperature, permeabilized using the Foxp3 Fixation/Permeabilization kit and stained with a fluorescein-conjugated anti-Foxp3 antibody. Data were collected using an LSRFortessa flow cytometer (BD Biosciences) and analyzed using FlowJo software (TreeStar).

### Real-time PCR

Lymphocytes cells isolated from lymph nodes were purified using flow cytometry, and RNA was extracted using TRIzol. cDNA was synthesized from the total RNA using oligo dT and SuperScriptⅢ reverse transcriptase (Invitrogen) and used as the template to amplify genes of interest and a housekeeping gene (GAPDH or 18S rRNA) by real-time PCR using the LightCycler480 Probe Master Mix kit (Roche 04707494001) in a LightCycler480 System (Roche Applied Science). mRNA levels were normalized to GAPDH using the comparative Ct method (ΔΔCt) or to 18S rRNA levels. The primer and probe sequences were as follows: 5'-GCTCCCCTGGTTACACTGAA-3' and 5'-ACGGGATACCGAGAAGCAT-3' for Ebi3 (probe #26); 5'-TCAGAATCACAACCATCAGCA-3' and 5'-CGCCATTATGATTCAGAGACTG-3' for P35 (probe #49); 5'-CATGGCATCACCTCTCTGAC-3' and 5'-AAGGGCCGAAGTGTGGTA-3' for P28 (probe #38); 5'-AGCTTGTCATCAACGGGAAG-3' and 5'-TTTGATGTTAGTGGGGTCTCG-3' for GAPDH (probe #9); and 5'-GCAATTATTCCCCATGAACG-3' and 5'-GGGACTTAATCAACGCAAGC-3' for 18S rRNA (probe #48).

### *In vitro* suppression assays

Treg suppression assays were performed as previously described with some modifications 22. Single-cell suspensions were prepared from the peripheral lymph nodes of 8-10 week-old wild-type congenic CD45.1 (BoyJ) mice as responders. The cells were preincubated for 10 min in 5 μM eFluor670 cell proliferation dye (eBioscience) in phosphate-buffered saline (PBS) before culture for analysis of proliferation by dye dilution Tr35 (CD4+YFP-CD62L^low^Thy1.1^+^) and Tconv (CD4^+^YFP^-^CD62L^low^Thy1.1^-^) cells from 35EbiT × Foxp3GFP-Cre-R26YFP triple transgenic mice (CD45.2) were sorted using FACS. *In vitro* Tr35 function was measured by culturing 1 × 10^5^ T responders with 1.5 μg/mL soluble anti-CD3 (145-2C11) and titrations of Tr35 cells in complete RPMI (RPMI 1640, 10% FCS, 2 mM L-glutamine, penicillin/streptomycin). The cells were cultured in a 96-well U-bottom plate at 37°C and 5% CO_2_ for 72 h. Wells with responder cells alone were used as positive controls. Finally, the cells were stained with anti-CD45.1, anti-CD4, anti-CD8, and eFluor506 (eBioscience) and analyzed by FACS on an LSRFortessa (BD Biosciences).

### *In vivo* T-cell labeling and Treg cell depletion

Mice were injected intravenously (i.v.) with biotin anti-CD4 (2 μg, RM4-4, eBioscience) and sacrificed 5 min later. Spleens were collected, and single-cell suspensions were prepared for flow cytometry analysis as described above. CD4 surface staining was performed using RM4-5 antibodies. For Treg cell depletion, Foxp3^DTR^ mice were injected i.p. with DT (Sigma, Saint Louis, MO, USA) at a dosage of 50 µg/kg of body weight. Control mice received phosphatebuffered saline (PBS).

### RNA sequencing

Lymphocytes were isolated from the lymph nodes of 35EbiT-Foxp3-DTR KI mice and enriched for CD4+ T cells by using anti-CD8α mAb (BioXcell), BioMag goat anti-Rat IgG (QIAGEN) and BioMag goat anti-mouse IgG beads (QIAGEN) according to the product protocol. Then, CD25^-^CD62L^low^Thy1.1^+^ and CD25^-^CD62L^hi^Thy1.1^-^ cells were sorted by FACS to a typical purity of >90% using Thy1.1, CD25, CD62L, and CD4 antibodies. RNA was extracted with RNAeasy mini kits according to the manufacturer's protocol (QIAGEN). The quantity of RNA was determined using a Nanodrop ND-1000 UV/Vis spectrophotometer (Thermo Fisher Scientific). The integrity of the RNA was verified using an Agilent 2100 Bioanalyzer and an RNA 6000 Nano Kit (Agilent Technologies, Palo Alto, CA). cDNA libraries were prepared using SMARTer amplification (Clontech). RNA sequencing (RNA-seq) and bioinformatics analysis were conducted by Novogene. The raw data (raw reads) in FASTQ format were processed using in-house Perl scripts. This step produced clean data (clean reads) by removing reads containing adapters, reads containing poly-N, and low-quality reads. The Q20, Q30, and GC contents of the clean data were calculated, and all downstream analyses were based on high-quality clean data. Reference genome and gene model annotation files were directly downloaded from http://ftp.ensembl.org/pub/release-77/gtf/mus_musculus/. Differential expression analysis of two conditions was performed using the DEGSeq R package (1.20.0). The p values were adjusted using the Benjamini-Hochberg method. A corrected q-value = 0.05 and log2(fold change) = 1 were set as the thresholds for significantly differential expression.

### Histology scoring

Hematoxylin and eosin (HE) staining and pathological scoring were conducted for histopathology. Lung tissue was fixed in 4% PFA, washed in PBS, and dehydrated in increasing concentrations of ethanol. Next, the tissue was embedded in paraffin, and 5-μm slices were prepared. The slices were deparaffinized, rehydrated, stained with HE according to the manufacturer's instructions, and analyzed by light microscopy. A 5-point scoring method was used to assess the severity of histopathological changes: 0 = no abnormality, 1 = minimal, 2 = mild, 3 = moderate, 4 = moderately severe, and 5 = severe. The cellular distribution was classified as focal, multifocal, or diffuse.

### Statistical analysis

FACS data were collected and processed using FACS analysis software (FlowJo). Data are presented as the mean ± SEM. Differences between data sets were analyzed by ANOVA with the Bonferroni post-test or an unpaired two-tailed Student's *t* test in Prism (GraphPad) software. P values of less than 0.05 were considered significant: *p < 0.05, **p < 0.01, and ***p < 0.001. p values >0.05 were considered not statistically significant (unmarked or specified as ns).

### Excel table

Table S1: Top 724 differentially expressed genes between Tr35 cells and Tconvs.

## Supplementary Material

Supplementary figures.

Table S1 Top 724 differential expression genes between Tr35 and Tconv
