# Supplementary material for: Adaptive plasticity of natural interleukin-35-induced regulatory T cells (Tr35) that are required for T-cell immune regulation
Source: Theranostics. 2024 May 5;14(7):2897–914. doi: 10.7150/thno.90608 (PMC11103508; doi:10.7150/thno.90608)
Supplement: Supplementary file 1 — Supplementary figures. [file thnov14p2897s1.pdf]

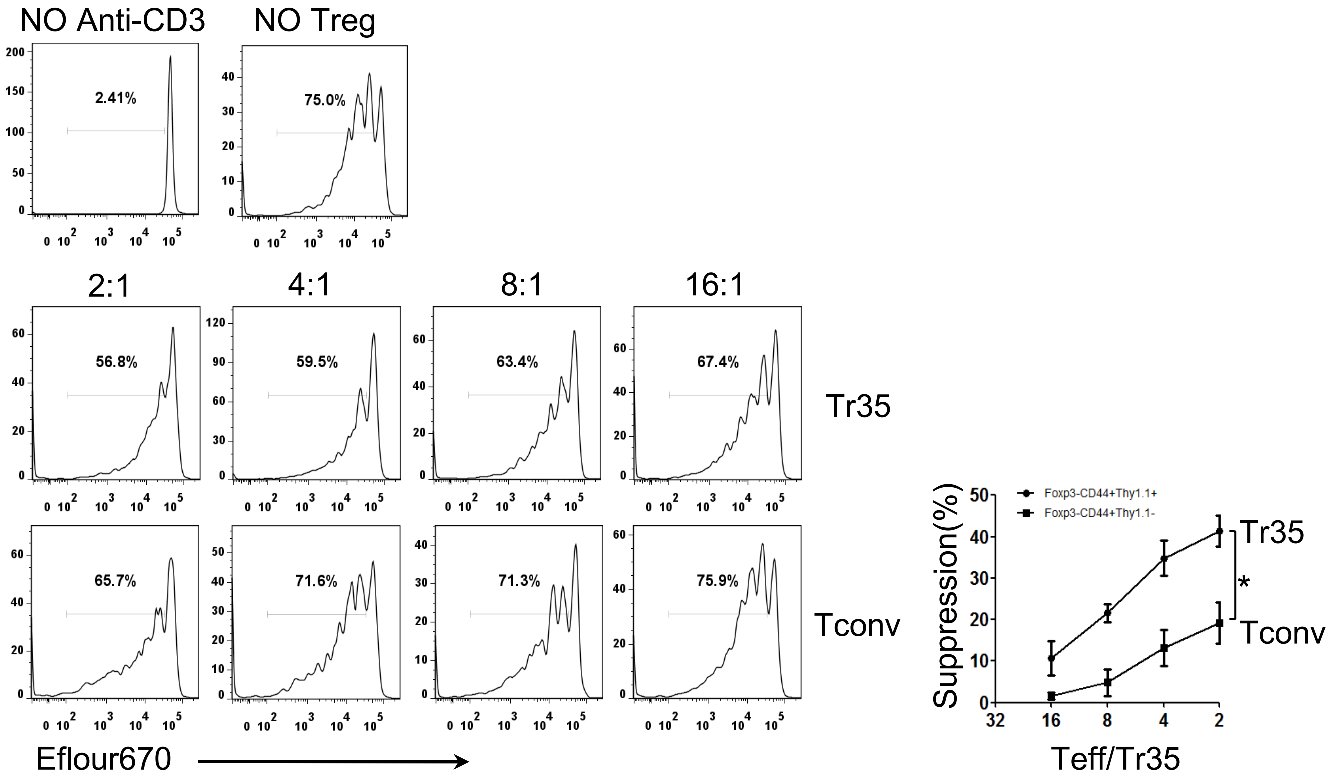

Figure S1. Functional analysis of Tr35 cells *in vitro*.

Proliferation of responder T cells cultured *in vitro* (horizontal axis) at various ratios with Tr35 ($YFP^{-}CD62L^{low}Thy1.1^{+}$) cells or Tconvs ($YFP^{-}CD62L^{low}Thy1.1^{-}$) sorted from the lymph nodes of 35EbiT × Foxp3GFP-Cre-R26YFP triple transgenic mice. The data shown are typical results from three similar experiments. *$p < 0.05$ (Student's t test).

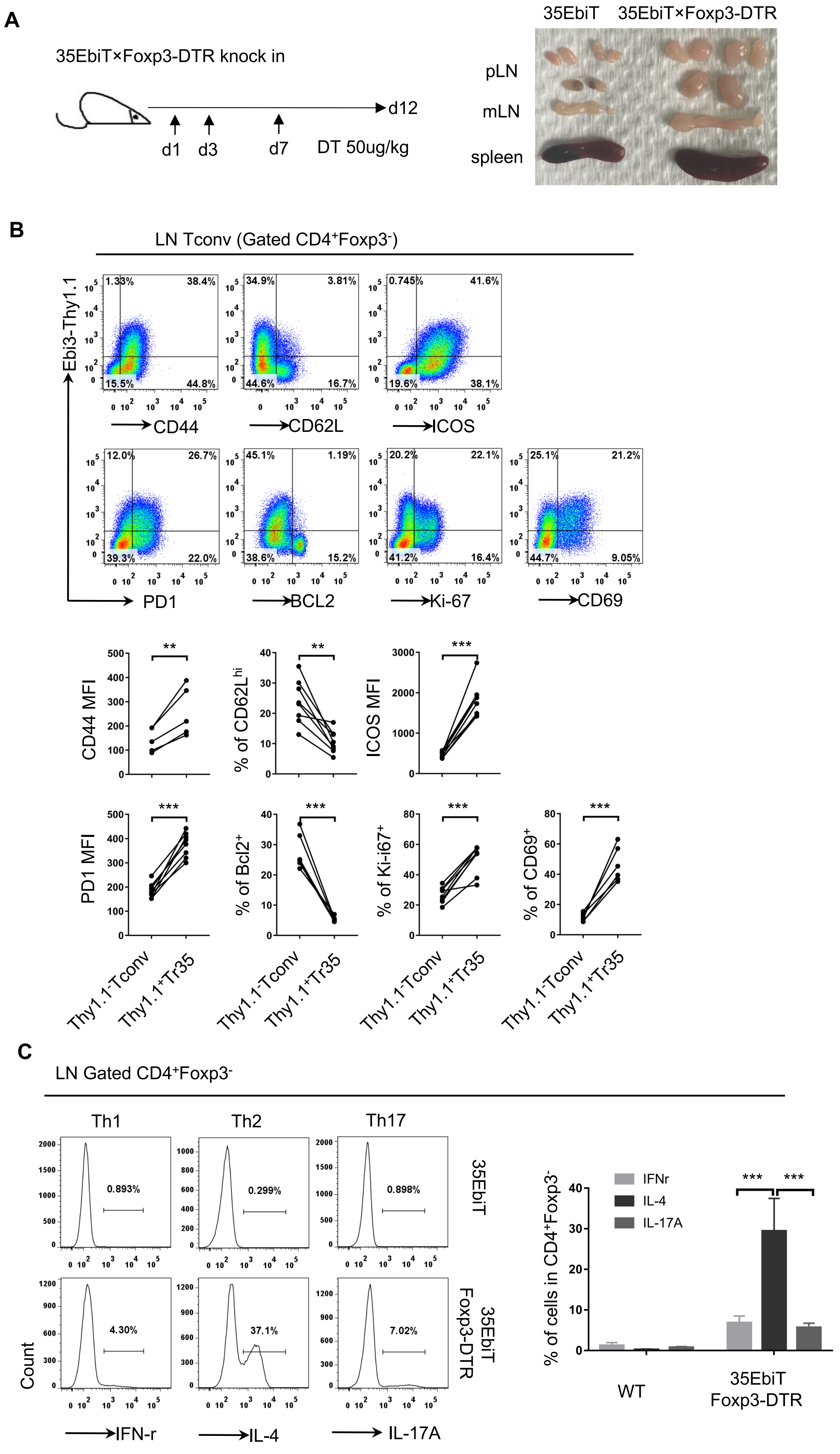
A
35EbiT×Foxp3-DTR knock in
d12
d1
d3
d7
DT 50ug/kg
35EbiT
35EbiT×Foxp3-DTR
pLN
mLN
spleen
B
LN Tconv (Gated CD4+Foxp3-)
Ebi3-Thy1.1
1.33%
38.4%
15.5%
44.8%
CD44
34.9%
3.81%
44.6%
16.7%
CD62L
0.745%
41.6%
19.6%
38.1%
ICOS
12.0%
26.7%
39.3%
22.0%
PD1
45.1%
1.19%
38.6%
15.2%
BCL2
20.2%
22.1%
41.2%
16.4%
Ki-67
25.1%
21.2%
44.7%
9.05%
CD69
CD44 MFI
% of CD62Lhi
ICOS MFI
PD1 MFI
% of Bcl2+
% of Ki-i67+
% of CD69+
Thy1.1-Tconv
Thy1.1+Tr35
C
LN Gated CD4+Foxp3-
Th1
Th2
Th17
0.893%
0.299%
0.898%
4.30%
37.1%
7.02%
35EbiT
35EbiT Foxp3-DTR
Count
IFN-r
IL-4
IL-17A
% of cells in CD4+Foxp3-
IFNr
IL-4
IL-17A
WT
35EbiT Foxp3-DTR

Figure S2. Phenotypic analysis of Tr35 cell subsets in 35EbiT × Foxp3-DTR KI reporter mice.

(**A**) Schematic representation of the experimental procedure. The picture compares the sizes of the lymph nodes and spleen from 35EbiT and 35EbiT × Foxp3-DTR KI reporter mice.

(**B**) Flow cytometry analysis of $CD4^+$ T cells isolated from the lymph nodes of 35EbiT-Foxp3-DTR transgenic mice 12 days after the third treatment with DT or PBS (control). The cells were stained with antibodies against CD4, Thy1.1, Foxp3, and other cell surface markers. The mean fluorescence intensity (MFI) and frequencies of the indicated markers reflect the cumulative results of two or three independent experiments, respectively.

(C) Flow cytometry analysis of the expression of IL-17A, IFN-γ and IL-4 by $CD4^+Foxp3^-Thy1.1^-$ Tconvs from the lymph nodes of DT-treated 35EbiT-Foxp3-DTR mice. The average frequency of cytokine-positive cells among gated subsets is shown.

The data shown are typical results from three similar experiments, n=5-7. The error bars represent the mean ± SEM. **p < 0.01 and ***p < 0.001 (A-B: Student's t test, C: analysis of variance (ANOVA) with Bonferroni post-test).

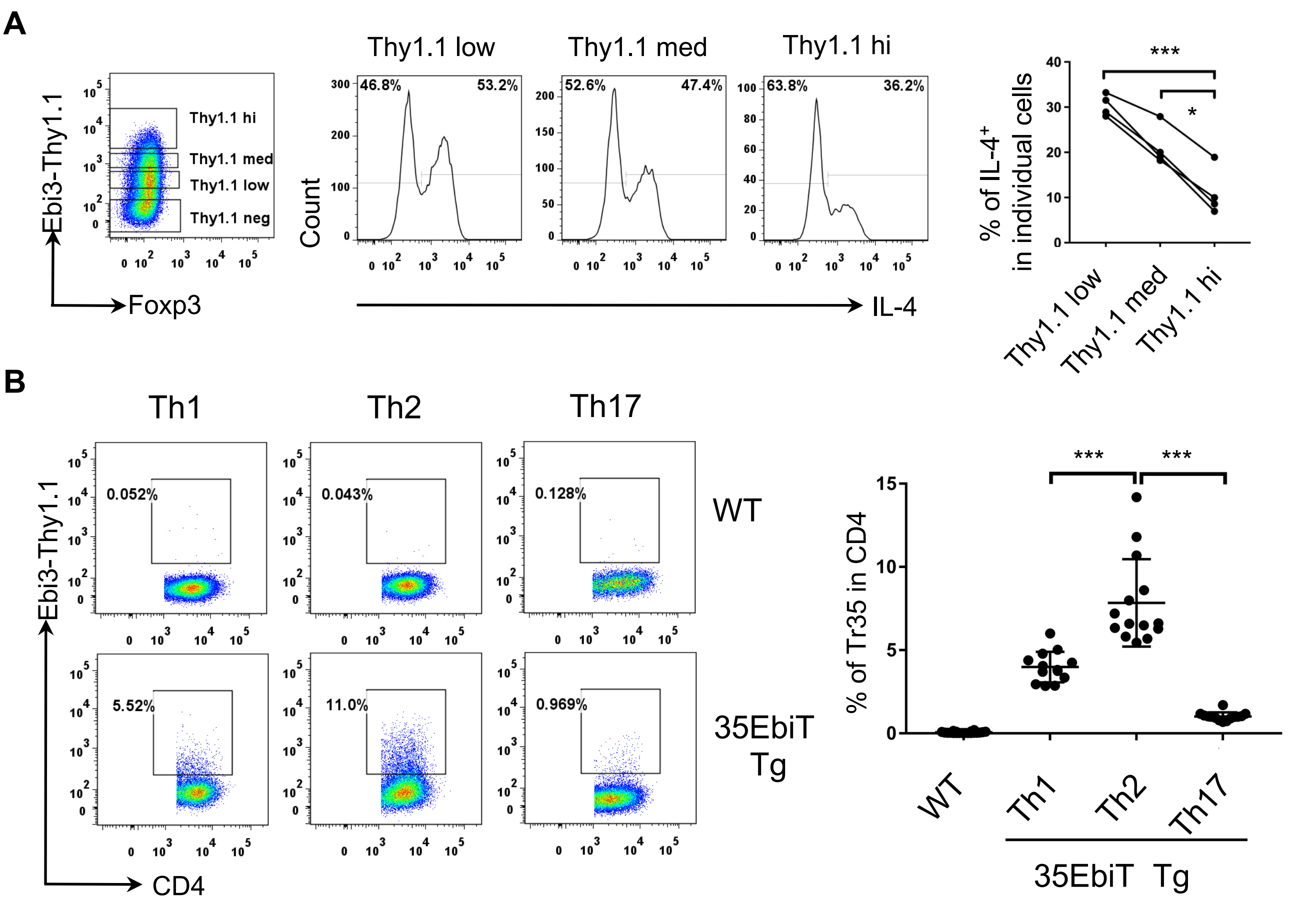

Figure S3. Induction of Tr35 cells *in vitro*.

(A) Flow cytometry analysis of cells from the lymph nodes of 35EbiT×Foxp3-DTR transgenic mice. The plot is gated on $CD4^+$ T cells with secondary gating on specific subsets based on Thy1.1 (Ebi3) expression levels. The numbers in the quadrants indicate the percentage of IL-4^+ cells in each gated subpopulation (lower right). The average frequencies of IL-4^+ cells in each gated subpopulation are shown.

(B) Magnetic bead-purified $CD4^+$ T cell fractions from 35EbiT mice or WT mice were cultured *in vitro* under various T-cell polarization conditions. The flow plots represent the expression of Thy1.1 (Ebi3) by $CD4^+$ T cells after 7 days of culture.

The data shown are typical results from two similar experiments, n=4-6. The error bars represent the mean ± SEM. **p < 0.01 and ***p < 0.001 (analysis of variance (ANOVA) with Bonferroni post-test).

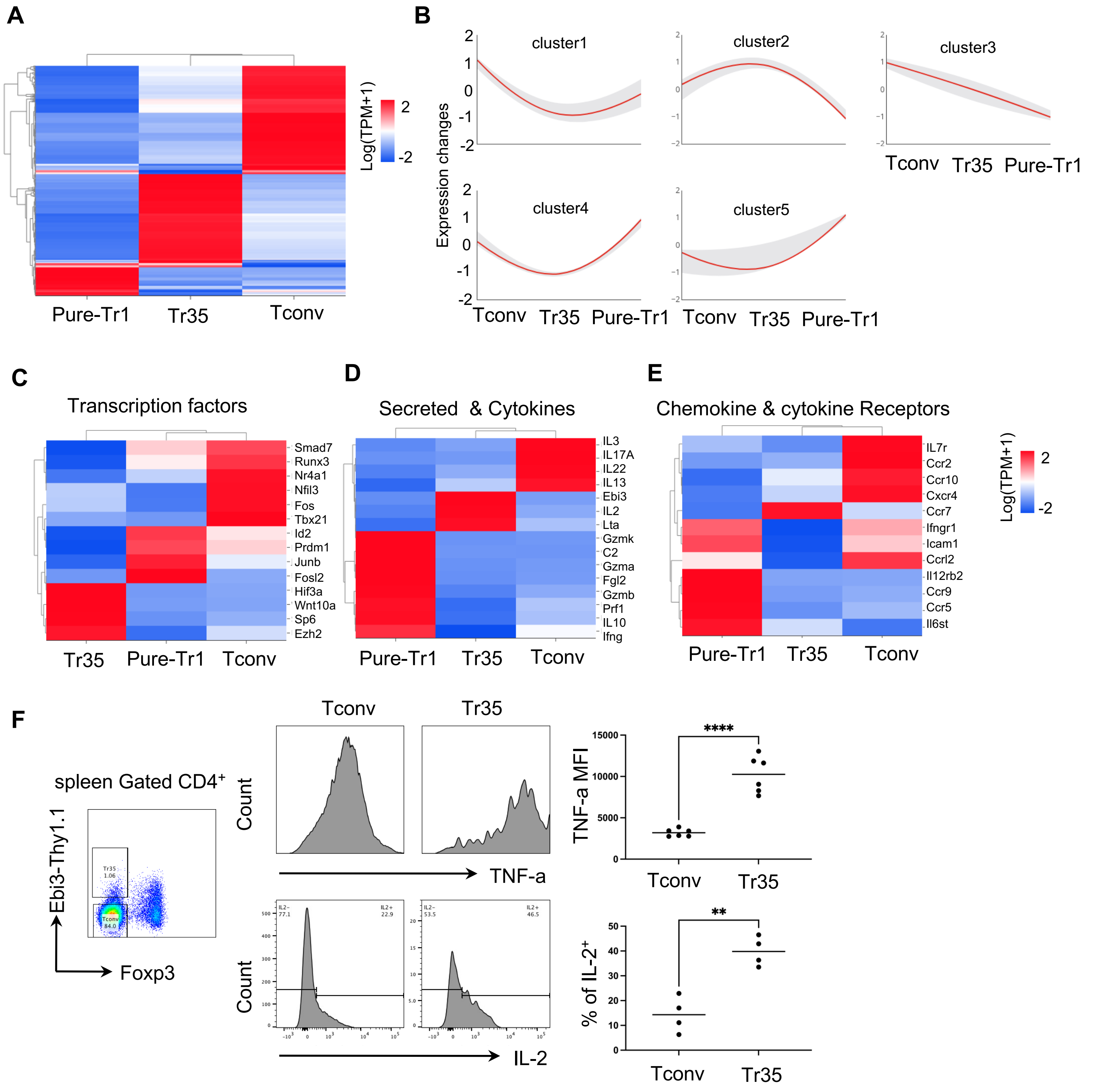

Figure S4. Transcriptional profiling of Tr35 and Tr1 cells

(**A**) Selected differentially expressed genes in Tr35 cells versus Tconvs plotted against those expressed in Pure-Tr1 cells [28]. The bioinformatics analysis was based on the genes listed in Supplementary Table 1.

(**B**) Relative mRNA expression pattern of the indicated cluster genes in Tr35 cells, Pure-Tr1 cells and Tconvs.

(**C-E**) Heatmap of genes selectively expressed in Tr35 cells, Pure-Tr1 cells or Tconvs.

(**F**) Flow cytometric analysis of Tr35 cell expression of IL-2 and TNF-α.

n=3; NES: normalized enrichment score; p. adj: adjusted p value; FDR: false discovery rate; (Student's t test).

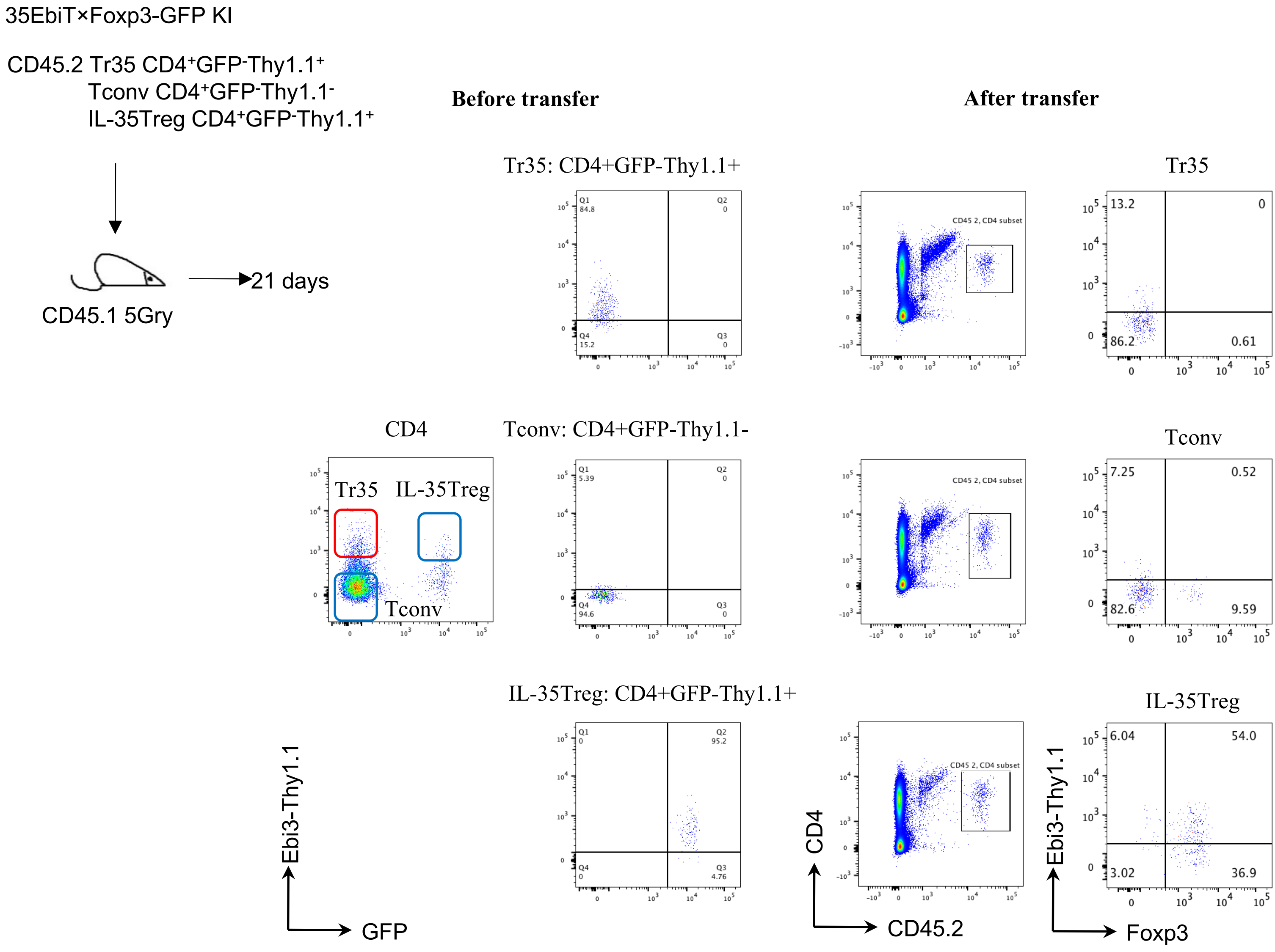

Figure S5. The plasticity of Tr35 and IL-35-Treg cells

We obtained Tr35 and IL-35-Treg cells through sorting and transferred them separately to mice irradiated with 5Gry. On day 21, we assessed the expression of Thy1.1 (Ebi3) in the transferred cells in the spleen.
